# STAT3 but Not STAT5 Contributes to the Protective Effect of Electroacupuncture Against Myocardial Ischemia/Reperfusion Injury in Mice

**DOI:** 10.3389/fmed.2021.649654

**Published:** 2021-07-09

**Authors:** Hui-Hui Guo, Xin-Yue Jing, Hui Chen, Hou-Xi Xu, Bing-Mei Zhu

**Affiliations:** ^1^Key Laboratory of Acupuncture and Medicine Research of Ministry of Education, Nanjing University of Chinese Medicine, Nanjing, China; ^2^Rehabilitation Medicine Department, YE DA Hospital of Yantai, Yantai, China; ^3^Regenerative Medicine Research Center, West China Hospital, Sichuan University, Chengdu, China

**Keywords:** myocardial ischemia reperfusion, STAT5, STAT3, electro-acupuncture, cardioprotection

## Abstract

Electroacupuncture (EA) can help reduce infarct size and injury resulting from myocardial ischemia/reperfusion (I/R); however, the underlying molecular mechanism remains unknown. We previously reported that STAT5 plays a critical role in the cardioprotective effect of remote ischemic preconditioning (RIPC). Here, we assessed the effects of electroacupuncture pretreatment (EAP) on myocardial I/R injury in the presence and/or absence of *Stat5* in mice and investigated whether EAP exerts its cardioprotective effects in a STAT5-dependent manner. Adult *Stat5*^*fl*/*fl*^ and *Stat5-*cKO mice were exposed to EAP at Neiguan (PC6) for 7 days before the induction of I/R injury by left anterior descending (LAD) coronary artery ligation. The myocardial infarct size (IS), area at risk, and apoptotic rate of cardiomyocytes were detected. RT-qPCR and western blotting were used to measure gene and protein expression, respectively, in homogenized heart tissues. RNA-seq was used to identify candidate genes and pathways. Our results showed that EAP decreased IS and the rate of cardiomyocyte apoptosis. We further found that STAT5 was activated by EAP in *Stat5*^*fl*/*fl*^ mice but not in *Stat5-*cKO mice, whereas the opposite was observed for STAT3. Following EAP, the levels of the antiapoptotic proteins Bcl-xL, Bcl-2, and p-AKT were increased in the presence of *Stat5*, while that of interleukin 10 (IL-10) was increased in both *Stat5*^*fl*/*fl*^ and *Stat5-*cKO. The gene expression profile in heart tissues was different between *Stat5*^*fl*/*fl*^ and the *Stat5-*cKO mice with EAP. Importantly, the top 30 DEGs under EAP in the *Stat5-*cKO mice were enriched in the IL-6/STAT3 signaling pathway. Our results revealed for the first time that the protective effect of EAP following myocardial I/R injury was attributable to, but not dependent on, STAT5. Additionally, we found that EAP could activate STAT3 signaling in the absence of the *Stat5* gene, and could also activate antiapoptotic, survival, and anti-inflammatory signaling pathways.

## Introduction

Electroacupuncture (EA) is based on acupuncture, a key component of traditional Chinese medicine. Numerous studies have demonstrated that EA is effective as an alternative protective treatment against myocardial ischemia/reperfusion (I/R) injury *via* electrical stimulation at specific acupoints (1–7). Recently, a clinical trial was undertaken to assess the effect of acupuncture treatment on a total of 1,651 patients with chronic stable angina. The results indicated that acupuncture, used as adjunctive therapy, could alleviate pain, reduce anxiety and depression, and improve the quality of life of the patients (5). Additionally, several studies have demonstrated the effectiveness of EA in treating cardiovascular diseases, and revealed some of the mechanisms underlying its effects. These include improving neurological function, modulating humor states (3, 8–11), regulating apoptosis (12–15), reducing calcium overload and antioxidative stress (16–19), activating anti-inflammatory pathways (12, 20), promoting angiogenesis (21), and regulating energy metabolism (22). However, the fundamental molecular mechanisms involved in the cardioprotective effect of EA have yet to be identified.

There is evidence that EAP can protect the ischemic myocardium against I/R injury (7, 14, 22, 23). While RIPC has been applied as one of common cardioprotective strategies against I/R injury (4, 24, 25), EAP is considered functionally similar to transcutaneous electrical nerve stimulation (TENS) and RIPC (4, 26). Studies on ischemic conditioning have been undertaken using different species, such as mice, rats, pigs, and humans (27–34). The most practical model of myocardial ischemia involves coronary occlusion, leading to the partial or complete obstruction of blood flow in a coronary artery, which mimics the clinical signs of coronary heart disease. Ideally, RIPC or EA pretreatment experiments on treating heart disease should be carried out using big animals or human patients as models (35, 36), whereas mechanistic studies are better performed on small animals, especially when a knockout model is needed. Additionally, evidence from both human patients and mice has indicated that STAT5 plays an important role in RIPC (27, 33, 37). Given that there are many similarities between EAP and RIPC, we therefore use the Stat5-knockout mice model to study the protection of EAP from myocardial I/R injury and its underlying mechanism.

We established a myocardial I/R mouse model using cardiomyocyte-specific *Stat5*-knockout (*Stat5-*cKO) mice. EA was applied to the mice 7 days before surgery to induce I/R injury. We also undertook genome-wide gene profiling to identify candidate genes involved in the cardioprotective role of EAP, and detected some functional pathways.

## Materials and Methods

### Mice

Stat5-floxed mice (*Stat5*^*fl*/*fl*^), generated as previously described (37), were a kind gift from Dr. Hennighausen (NIDDK, NIH). *Tnnt2*-Cre male mice (*Tnnt2Cre*) were a gift from Bin Zhou (Shanghai Institutes for Biological Sciences of the Chinese Academy of Sciences). *Stat5-*cKO mice were generated by crossing these two genotypes. Doxycycline hyclate (Sigma-Aldrich) was added to the drinking water of mice at a concentration of 2 mg/mL and administered for 7 days. Genotyping was performed as previously described (37).

### Study Groups

The mice were randomly divided into the following four groups: *Stat5*^*fl*/*fl*^+I/R, *Stat5*^*fl*/*fl*^+EA+I/R, *Stat5-*cKO+I/R, and *Stat5-*cKO+EA+I/R. The *Stat5*^*fl*/*fl*^+I/R and *Stat5-*cKO+I/R mice were exposed to LAD coronary artery occlusion for 30 min, and then reperfused for 180 min, while the *Stat5*^*fl*/*fl*^+EA+I/R and *Stat5-*cKO+EA+I/R mice were subjected to EAP treatment 7 days before LAD artery ligation. All animal studies were carried out according to Chinese and international guidelines for the experimental use of animals. All experiments were approved by the Institute for Animal Care and Use Committee at Nanjing University of Chinese Medicine.

### *In vivo* Experiments

EA was performed at bilateral PC6 (also called Neiguan) acupoints in the *Stat5*^*fl*/*fl*^+EA+I/R and *Stat5-*cKO+EA+I/R mice as previously described (12). The PC6 acupoints are located in the anteromedial aspect of the forelimb between the radius and ulna, 3-mm proximal to the wrist joints (12, 38). Anesthesia was induced with 5% isoflurane and maintained with 1–2% isoflurane in pure oxygen. Sterilized disposable stainless steel acupuncture needles (0.18 mm ×13 mm, Beijing Zhongyan Taihe Medical Instruments Factory, Beijing, China) were inserted into the muscle layer ~1–2 mm below bilateral PC6 simultaneously using Han's EA instrument (Han Acuten, WQ1002F, Beijing, China). The frequency was 2/15 Hz (alternating dense and disperse mode) and the intensity was 0.5–1.0 mA. Stimulation was applied for 20 min once a day for a total of 7 days. The mice in the *Stat5*^*fl*/*fl*^+I/R and the *Stat5-*cKO+I/R groups were restrained for 20 min without EA stimulation. The *Stat5*^*fl*/*fl*^+I/R and *Stat5-*cKO+I/R mice (control groups) were also anesthetized daily for 7 days before I/R surgery.

The I/R operation was performed according to a previously described protocol (12, 37). Briefly, all the mice were anesthetized by 5% isoflurane and anesthesia was then maintained with 2% isoflurane in a mixture of 70% N_2_O and 30% O_2_. Under anesthesia, the mice were subjected to a left thoracotomy and LAD artery ligation. Ischemia was confirmed by myocardium blanching, as well as ST-segment elevation and widening of the QRS complex in ECG (37). After 30 min, reperfusion was performed by quickly releasing and removing the suture continuously for 3 h. In the sham-operation group, the same procedure was performed except for the LAD artery ligation. Mice were euthanized by cervical dislocation and the heart specimens were harvested.

### Determination of Infarct Size

After harvesting, the hearts were injected for 1–2 min with 0.2 mL of 2% Evans blue dye into the ventricle as previously described (39, 40). The excised and frozen hearts were quickly sliced into five pieces, placed in 2 mL of 1% TTC (Sigma-Aldrich, St. Louis, MO, USA) in phosphate-buffered saline (PBS), and incubated at 37°C for 15 min. After incubating, the sections were placed in 4% (*v*/*v*) paraformaldehyde at 4°C for 12 h. Unaffected myocardial tissue was stained blue, while the area at risk (AAR) and the infarcted area were unstained and showed as red or white. The infarcted area, AAR, and total left ventricular (LV) area were quantified using Image-Pro Plus 6.0 software (NIH, USA). The infarct size (IS) and AAR percentages were calculated using the following formulas: IS (%) = IS/AAR ×100; AAR (%) = AAR/total LV area ×100 (39, 40).

### Apoptosis Measurements

TUNEL staining was used to detect cell apoptosis in cardiac tissue in each group. All the protocols were performed as previously described (37). Heart tissues were harvested and embedded in optimal cutting temperature (OCT) compound (Thermo Scientific, USA). Then, 8-μm-thick tissues were subjected to TUNEL staining according to the instructions of the manufacturer (Cat no. 11684817910, Roche Diagnostics, Lewes, UK). Ten sections were randomly selected from at least 3 animals per group and visualized using a fluorescence microscope (Nikon, Japan). DNase-I served as the positive control labeling solution as the negative control.

### Western Blotting

Whole-ventricle samples were lysed with RIPA buffer containing protease and phosphatase inhibitors based on the Protease Inhibitor Cocktail (100X) (Thermo Scientific, USA). Homogenates were centrifuged at 14,000 × g for 10 min at 4°C, and the collected supernatants were stored at −80°C until further use. Protein concentrations were determined using a BCA protein assay (Pierce, Thermo Scientific, USA). Protein was mixed with 5 × Laemmli loading buffer and heated at 95°C for 10 min. Equal amounts of protein were subjected to SDS–PAGE and transferred to polyvinylidene fluoride membranes. The samples were incubated with primary antibodies against Bcl-xL (1:1,000, Cell Signaling Technology, #2762), Bcl-2 (1:1,000, Cell Signaling Technology, #3498), Cyt c (1:1,000, Cell Signaling Technology, #4280), phospho-STAT5 (1:1,000, Cell Signaling Technology, #4322), STAT5 (1:1,000, Cell Signaling Technology, #94205), phospho-STAT3 (1:1,000, Cell Signaling Technology, #4093), STAT3 (1:1,000, Cell Signaling Technology, #4904), phospho-AKT (1:1,000, Cell Signaling Technology, #4060), AKT (1:1,000, Cell Signaling Technology, #4298), IL-10 (1:1,000, Abcam, #ab192271), VEGFA (1:1,000, Abcam, #ab46154), beta-actin (1:1,000, Abcam, #ab8226), or GAPDH (1:1,000, Cell Signaling Technology, #2118) overnight at 4°C, and then with a secondary antibody for 2 h at room temperature. Immunoreactive bands were revealed using SuperSignal West Pico Chemiluminescent Substrate (Pierce) and quantified using the ChemiDoc Imaging System (Bio-Rad).

### Quantitative Reverse Transcription PCR

Total RNA was extracted from heart tissue using TRIzol reagent (Invitrogen, Mannheim, Germany), and reverse-transcribed to cDNA using reverse transcriptase and random primers (11121ES60, Yeasen Biotech Co., Ltd., China). Target genes were amplified on a MX3000P thermocycler (Stratagene, La Jolla, CA, USA) using SYBR Green (Q431-02, Vazyme Co., China). Gene expression was quantified using the 2^−ΔΔCt^ method. The primer sequences were as follows: *Il6*, GACTTCACAGAGGATACCACCC (forward) and GACTTCACAGAGGATACCACCC (reverse); *gp130*, GAGCTTCGAGCCATCCGGGC (forward) and AAGTTCGAGCCGCGCTGGAC (reverse); beta-actin, GGTGAAGACGCCAGTAGAC (forward) and TGCTGGAAGGTGGACAGTGA (reverse).

### RNA Sequencing Analysis

RNA-seq for mouse heart tissues was performed using the Illumina Hiseq 2500 and 2000 platform (Illumina, USA) as described in our previous study (41). Data analysis was performed as previously described (41). The quality of the raw sequencing data was assessed by FastQC. The Cufflinks and Cuffdiff programs were used to assemble individual transcripts and for differential transcript expression analysis, respectively. The pathways were analyzed using DAVID Bioinformatics Resources. Genes with fewer than 1.0 fragments per kilobase of exon per million fragments mapped (FPKM) were filtered out. Log2 fold change (FC) ≥| ±1| and *P* < 0.05 were used as thresholds for identifying upregulated and downregulated genes.

### Statistical Analysis

Data analyses and treatment conditions were double-blinded. SPSS 18.0 software was used for statistical analysis. All data were expressed as means ± standard error of the mean (SEM). A two-tailed unpaired Student's *t*-test was used for comparisons between two groups. For comparisons between multiple groups, one-way or two-way ANOVA was used followed by the Bonferroni *post-hoc* test when equal variances were assumed. *P* < 0.05 was considered statistically significant.

## Results

### EAP Reduced Myocardial Infarct Size and Attenuated Cardiomyocytic Apoptosis to the Same Extent in Both *Stat5^*fl*/*fl*^* and *Stat5-*cKO Mice

**Figure 1:**
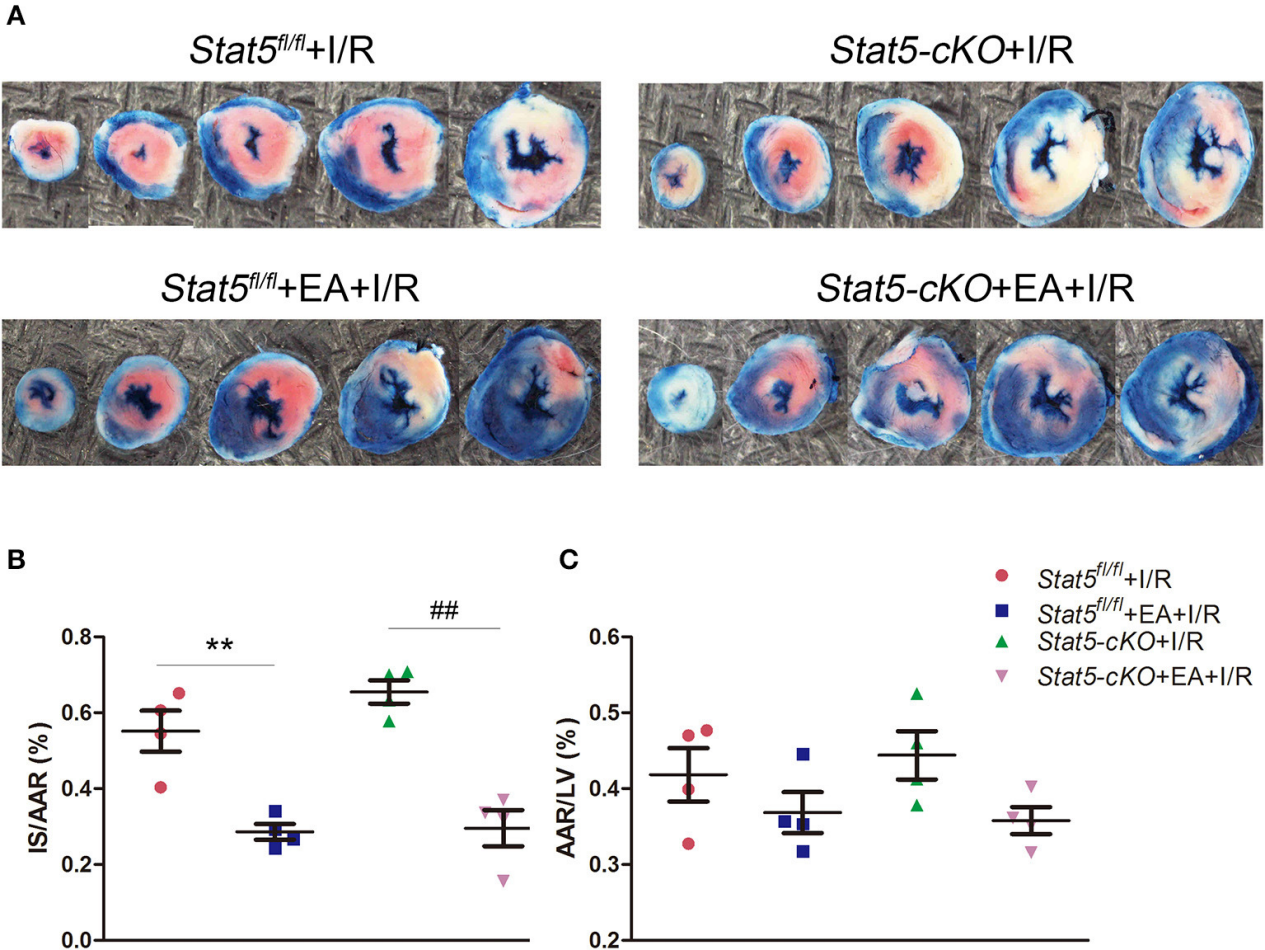
Acupuncture reduced myocardial infarct size. **(A)** Evans blue/TTC double staining was used to measure the ischemic infarct area and area at risk (AAR). **(B)** The infarct size (IS)/AAR ratio was calculated and presented as a percentage. Data are presented as means ± SEM. Normal tissues are stained blue, ischemic infarct areas and AARs are pale white or red. **P < 0.01 compared with *Stat5*^*fl*/*fl*^+I/R group; ^##^*P* < 0.01 compared with *Stat5-*cKO+I/R group. **(C)** The ratio of AAR/total left ventricular (LV) area was calculated and presented as a percentage. There was no difference between the groups. Data were analyzed by one-way ANOVA with Tukey's *post-hoc* correction, *n* = 4.

EAP had no effect on the daily behavior or cardiac performance of mice of either genotype. After myocardial I/R surgery, we harvested the heart tissues and measured myocardial infarct areas and AARs (Figure 1). We found that EAP significantly reduced infarct size in both *Stat5*^*fl*/*fl*^ mice (55.2 ± 10.8% without EAP vs. 28.6 ± 4.1% with EAP, *P* < 0.01) and *Stat5-*cKO mice (65.5 ± 5.3% without EAP vs. 29.6 ± 9.6% with EAP, *P* < 0.01). No significant difference in AAR was seen between the *Stat5*^*fl*/*fl*^+EA+I/R and the *Stat5-*cKO+EA+I/R mice.

**Figure 2:**
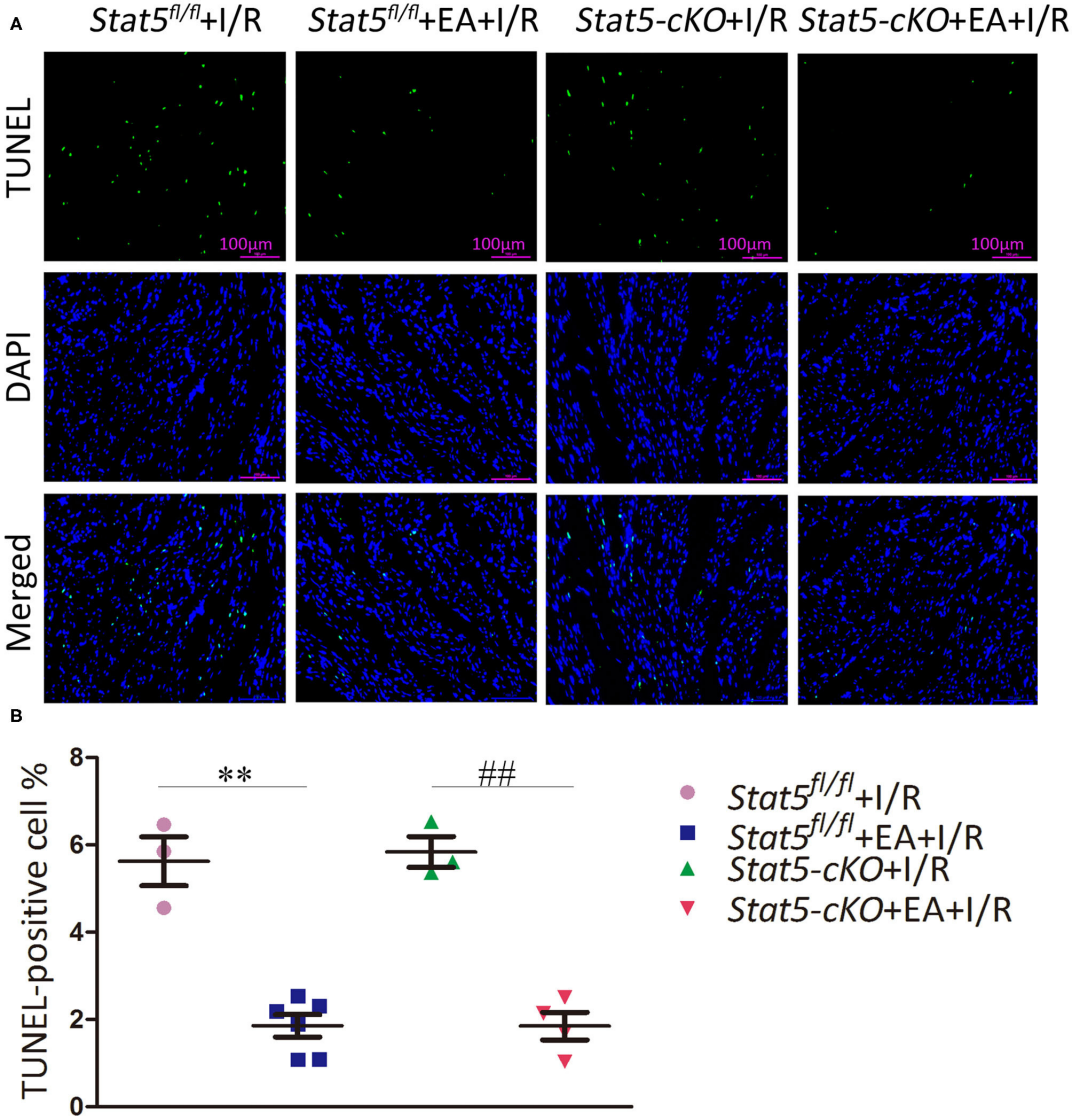
The effects of electroacupuncture pretreatment (EAP) on apoptosis in myocardial tissues in the *Stat5*^*fl*/*fl*^ and the *Stat5-*cKO mice. **(A,B)** The rate of apoptosis was measured by TUNEL staining. Values are means ± SEM. **P < 0.01 compared with the *Stat5*^*fl*/*fl*^+I/R group; ^##^*P* < 0.01 compared with the *Stat5-*cKO+I/R group. Data were analyzed by two-way ANOVA with Bonferroni's multiple comparison test, *n* = 3–6.

TUNEL staining was performed to detect apoptosis in myocardial cells. As shown in Figure 2, mice in the *Stat5*^*fl*/*fl*^+EA+I/R group had fewer TUNEL-positive cells compared with those in the *Stat5*^*fl*/*fl*^+I/R group (1.85 ± 0.26% vs. 5.62 ± 0.56%, *P* < 0.01). Similarly, the number of apoptotic myocardial cells was significantly lower in mice of the *Stat5-*cKO+EA+I/R group than in those of the *Stat5-*cKO+I/R group (1.85 ± 0.32 vs. 5.83 ± 0.35%, *P* < 0.01).

### EAP Activated STAT5 in *Stat5^*fl*/*fl*^* Mice, but Not in *Stat5-*cKO Mice, Following Myocardial I/R Surgery

**Figure 3:**
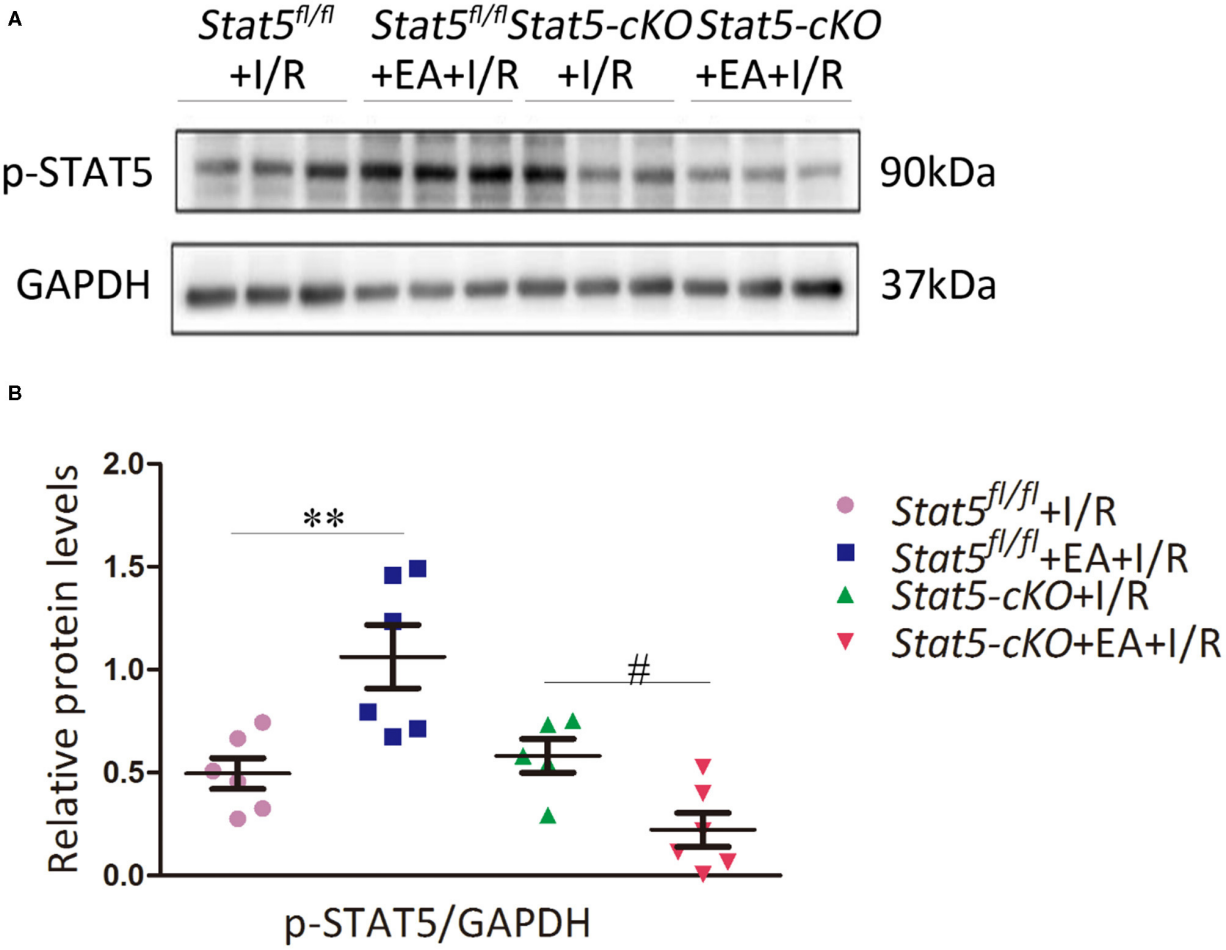
Electroacupuncture pretreatment (EAP) activated the STAT5 protein in the heart tissues of *Stat5*^*fl*/*fl*^ mice, but not in those of *Stat5-*cKO mice. **(A)** Representative western blotting images. **(B)** Quantitative analysis of p-STAT5 protein levels in each group. Data are presented as means ± SEM of at least three independent experiments. **P < 0.01 compared with the *Stat5*^*fl*/*fl*^+I/R group; ^#^*P* < 0.05 compared with the *Stat5-*cKO+I/R group. Data were analyzed by two-way ANOVA with Bonferroni's multiple comparison test, *n* = 6.

To further explore whether the myocardial protective effect of EAP against I/R injury is STAT5-dependent, we examined the expression of p-STAT5 in heart tissues by western blotting. EAP markedly increased the protein levels of p-STAT5/GAPDH in *Stat5*^*fl*/*fl*^ mice compared with those in *Stat5*^*fl*/*fl*^ mice subjected to I/R; however, EAP did not affect STAT5 activation in the hearts of *Stat5-*cKO mice (Figures 3A,B). This suggested that STAT5 may be involved in the EAP-mediated protective effects against myocardial I/R injury.

### EAP Activated IL-6/gp130/STAT3 Signaling in the Absence of *Stat5*

**Figure 4:**
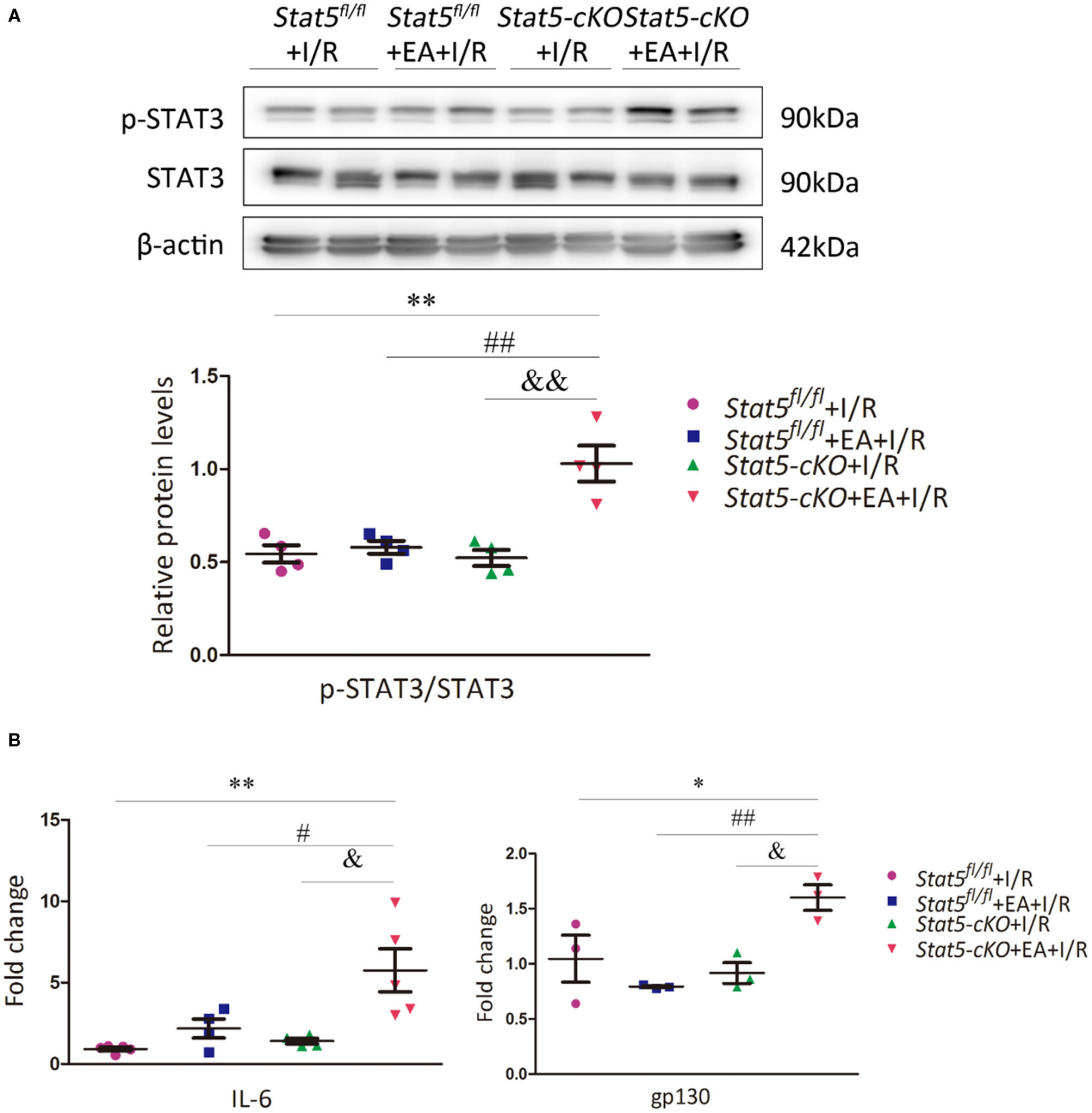
The expression of IL-6/gp130/STAT3 axis-related molecules. **(A)** The protein expression of STAT3 and p-STAT3 was assessed by western blotting. **P < 0.01 compared with the *Stat5*^*fl*/*fl*^+I/R group; ^##^*P* < 0.01 compared with the *Stat5*^*fl*/*fl*^+EA+I/R group; ^&&^*P* < 0.01 compared with the *Stat5-*cKO+I/R, *n* = 4. **(B)** The expression of *Il6* and *gp130* mRNA was measured by RT-qPCR. Data are presented as means ± SEM of at least three independent experiments. *P < 0.05, **P < 0.01 compared with the *Stat5*^*fl*/*fl*^+I/R group; ^#^*P* < 0.05, ^##^*P* < 0.01 compared with the *Stat5*^*fl*/*fl*^+EA+I/R group; ^&^*P* < 0.05 compared with the *Stat5-*cKO+I/R group. Data were analyzed by two-way ANOVA with Bonferroni's multiple comparison test, *n* = 3–5.

Given that STAT3 might compensate for the loss of STAT5, we then evaluated the STAT3 and p-STAT3 protein expression levels in the heart tissues of both *Stat5*^*fl*/*fl*^ and *Stat5-*cKO mice. The results showed that the expression of p-STAT3 was increased in *Stat5-*cKO+EA+I/R mice compared with that in mice of the *Stat5-*cKO+I/R group; however, this was not observed in *Stat5*^*fl*/*fl*^ mice (Figure 4A). To understand the mechanism by which STAT3 was activated in this process, we further assessed the expression levels of genes acting upstream of STAT3. We found that the mRNA expression of *Il6* and *gp130* was greatly increased in *Stat5-*cKO+EA+I/R mice compared with that in *Stat5-*cKO+I/R mice; however, these effects were not observed in the presence of *Stat5*. This suggested that, in the absence of the *Stat5* gene, EAP may activate the IL-6/gp130/STAT3 pathway at the mRNA level when the heart is exposed to myocardial I/R injury (Figure 4B).

### Genome-Wide Analysis Revealed the Gene Expression Profiles in Both *Stat5^*fl*/*fl*^* and *Stat5-*cKO Mice With or Without EAP Followed by Myocardial I/R Injury

**Figure 5:**
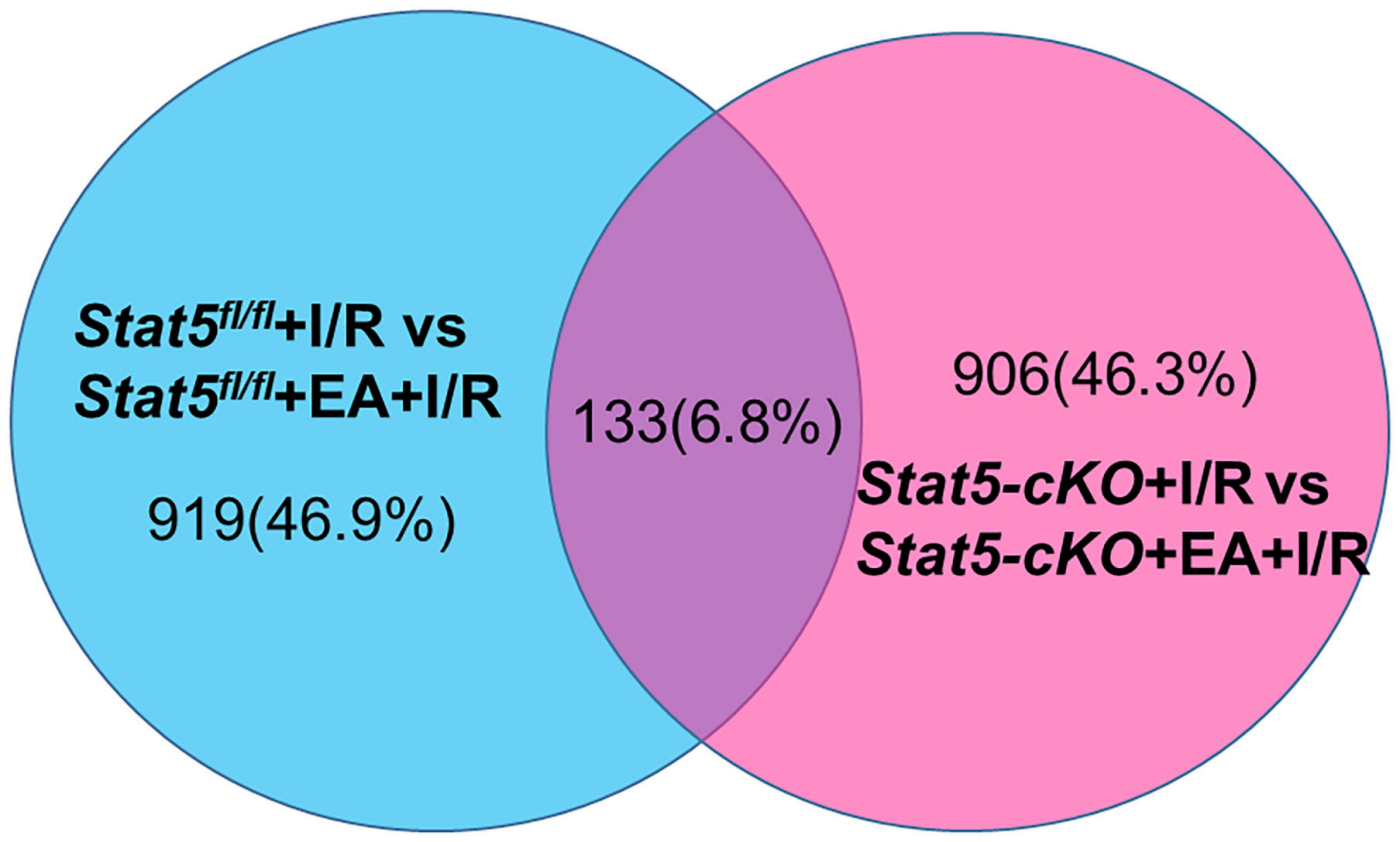
Venn diagrams and clustering analysis of RNA-seq results. Venn diagrams were drawn based on the RNA-seq datasets. The blue circle indicates the numbers of up- and downregulated genes in the *Stat5*^*fl*/*fl*^+EA+I/R group *vs*. the *Stat5*^*fl*/*fl*^+I/R group the pink circle represents the numbers of up- or downregulated genes in the *Stat5-*cKO+EA+I/R group *vs*. the *Stat5-*cKO+I/R group. A total of 133 genes overlapped between these two clusters.

To identify genes that may have a role in EAP-mediated protection against myocardial I/R injury, RNA was extracted from the heart tissues for RNA-seq analysis. The Cufflinks package was used to filter out the top 30 differentially expressed genes (DEGs) between the *Stat5*^*fl*/*fl*^+I/R and *Stat5*^*fl*/*fl*^+EA+I/R groups and the *Stat5-*cKO+I/R and *Stat5-*cKO+EA+I/R groups (Tables 1A,B). Venn diagrams were drawn based on the list of filtered DEGs among these four groups (Figure 5). The results showed that 1,052 genes were differentially expressed between the *Stat5*^*fl*/*fl*^+I/R and *Stat5*^*fl*/*fl*^+EA+I/R groups, while 1,039 genes were found to be differentially expressed between the *Stat5-*cKO+I/R and *Stat5-*cKO+EA+I/R groups; of these DEGs, 133 overlapped between these two clusters (Figure 5). Among the four groups, only two genes, *Hspa1a* and *Pttg1*, were found to be differentially expressed in all the groups (Table 1).

**Table 1:** The top 30 differentially expressed genes with a log2 (FC) > |±1|and *q* < 0.05.

**Up-regulated in EA against I/R**				**Down-regulated in EA against I/R**			
**Gene name**	**Value_1**	**Value_2**	**log2 (fold_change)**	**Gene name**	**Value_1**	**Value_2**	**log2 (fold_change)**
**A**. The top 30 differentially expressed genes obtained from comparing *Stat5^*fl*/*fl*^+*EA+I/R vs. *Stat5^*fl*/*fl*^*+I/R							
Fosb Retnlg Crisp1 Fos Cxcl5 Selp Cxcl1 S100a8 Atf3 Ptx3 Nr4a3 Sele Socs3 Egr1 S100a9 Il18rap Thbs1 Rdh12 Hspa1b Hspa1a Adam8 Ch25h Nts Ifitm6 Egr2 Arc Agt Rnd1 Pdk4 Plaur	0.181422 1.26857 1.13838 1.59922 0.375871 0.314312 1.96399 5.82634 2.46681 0.51755 0.472838 0.190666 2.33823 3.52759 11.0427 0.0722091 1.59068 0.122831 2.40171 1.99294 0.400678 0.769351 0.799365 1.20354 0.16011 1.14086 1.55521 0.793702 35.6426 1.59226	27.5826 106.281 82.4984 114.642 21.1825 16.2764 95.2442 256.694 106.299 19.8976 17.3526 6.83815 83.0272 122.674 381.325 2.21294 41.5123 3.01114 58.418 44.6613 8.43168 15.8707 15.3258 22.6423 3.01005 20.7638 27.442 12.8902 578.857 25.5567	7.24827 6.38853 6.17931 6.16363 5.81649 5.69443 5.59977 5.46131 5.42933 5.26476 5.19767 5.16449 5.1501 5.12 5.10986 4.93764 4.70582 4.61557 4.60428 4.48606 4.39531 4.36658 4.26096 4.23367 4.23265 4.18587 4.1412 4.02154 4.02153 4.00455	Hbb-bt Tcf15 Ccn5 Myl4 Scand1 Zhx2 Nrtn Tnfrsf25 Pttg1 Zfp771 Fzd2 Fxyd3 Aplnr Cited4 Eva1b Msx1 Dkk3 Rnaset2a Ifi27l2a Nrarp Kctd15 Hic1 Gas1 Oas1a Dynll1 Trim47 Tmsb10 B3gnt3 Myo7a Fam181b	35.5975 53.5841 6.67781 60.998 105.37 1.93407 43.6184 3.90054 33.8997 19.5158 3.1422 3.78128 15.2807 17.5033 18.5641 3.34857 5.70321 22.5668 180.402 11.2641 2.65098 7.47804 16.0842 3.5286 78.6361 26.4865 110.513 2.39685 2.29499 3.87361	1.35656 3.7788 0.624545 5.99943 11.7828 0.220615 5.0225 0.503182 4.94227 3.02169 0.491571 0.621019 2.89142 3.57884 3.80599 0.6924 1.21389 4.88218 39.5256 2.52363 0.611701 1.74196 3.82293 0.843947 18.9174 6.40037 26.7491 0.580215 0.574977 0.976908	−4.71375 −3.82581 −3.4185 −3.34586 −3.16071 −3.13204 −3.11846 −2.95452 −2.77803 −2.69122 −2.6763 −2.60617 −2.40186 −2.29006 −2.28617 −2.27387 −2.23213 −2.2086 −2.19036 −2.15815 −2.11563 −2.10195 −2.07289 −2.06387 −2.05548 −2.04903 −2.04665 −2.04648 −1.99691 −1.98738
**B**. The top 30 differentially expressed genes obtained from comparing *Stat5-cKO+*EA+I/R vs. *Stat5-cKO*+I/R.							
Eno1b Dynlt1b H2-Q1 Tmem181c-ps Gm4737 2610005L07Rik Gm14421 Mmp3 Gm42887 Ubb Hba-a2 Tmem191c Gdnf Rpl3-ps1 Stbd1 Adh6b Hba-a1 Polr2l Myh7 Gapdh Pttg1 Zc3h3 Gm6472 Cys1 Tgtp2 Rps6 Hspa1a Eif3j2 Gm8116 Gm15459	0.503646 1.11668 0.264109 0.99792 0.445121 1.25086 0.377049 0.379936 0.544175 35.8823 44.9947 0.28056 0.160275 3.02696 0.465139 0.422625 78.1547 6.80429 174.331 603.792 5.6626 0.178519 5.6958 0.799506 0.951374 94.127 20.8766 0.89728 1.05436 34.4931	6.53728 14.442 3.03007 11.1342 4.58914 12.1968 3.17075 3.17412 4.41682 290.433 363.254 2.17679 1.21115 22.6124 3.32577 3.00224 542.904 43.8946 1091.89 3577.93 32.9153 1.03346 31.1981 4.37525 5.16733 497.199 109.614 4.63881 5.39728 176.148	3.69821 3.69299 3.52015 3.47994 3.36595 3.28551 3.072 3.06253 3.02087 3.01686 3.01315 2.95582 2.91776 2.90117 2.83795 2.82859 2.79629 2.68952 2.64692 2.567 2.53922 2.53333 2.45349 2.45218 2.44134 2.40114 2.39248 2.37012 2.35587 2.35241	Olfr1033 Gm45551 Gm38271 Psg16 Gm3365 Sugct Gm43197 Gm15280 CAAA01147332.1 Zfp729a Adgra3 Fmod Dpy19l3 Gm37324 Gm48274 Pilra Prc1 Clec4e Spp1 Rac2 Oxnad1 Fggy Gm44215 Lars2 Zfp975 Bace2 Suds3 Tesk1 Insig2 Pnkp	855.24 221.558 30.2569 2.41103 7.26917 11.7515 55.389 32.1097 97.5278 6.97773 25.8651 3.18759 2.2277 1.86878 72.516 3.10358 1.51292 6.2329 3.08682 14.0493 117.772 4.18537 2.03074 129.273 3.23415 8.13204 101.31 11.7699 86.0597 30.3154	3.24857 1.31604 0.353232 0.0719611 0.262255 0.52977 3.11501 2.05499 7.86365 0.621896 3.26536 0.452927 0.347531 0.299513 11.6454 0.507 0.247267 1.08171 0.543414 2.49669 21.1786 0.772849 0.376049 25.3961 0.636673 1.6647 22.7191 2.74535 20.4798 7.36189	−8.04038 −7.39534 −6.4205 −5.06629 −4.79275 −4.47133 −4.15229 −3.96581 −3.63254 −3.48801 −2.98569 −2.81512 −2.68034 −2.64141 −2.63854 −2.61387 −2.6132 −2.52658 −2.506 −2.49241 −2.47532 −2.4371 −2.43301 −2.34774 −2.34476 −2.28836 −2.1568 −2.10004 −2.07113 −2.0419

**Figure 6:**
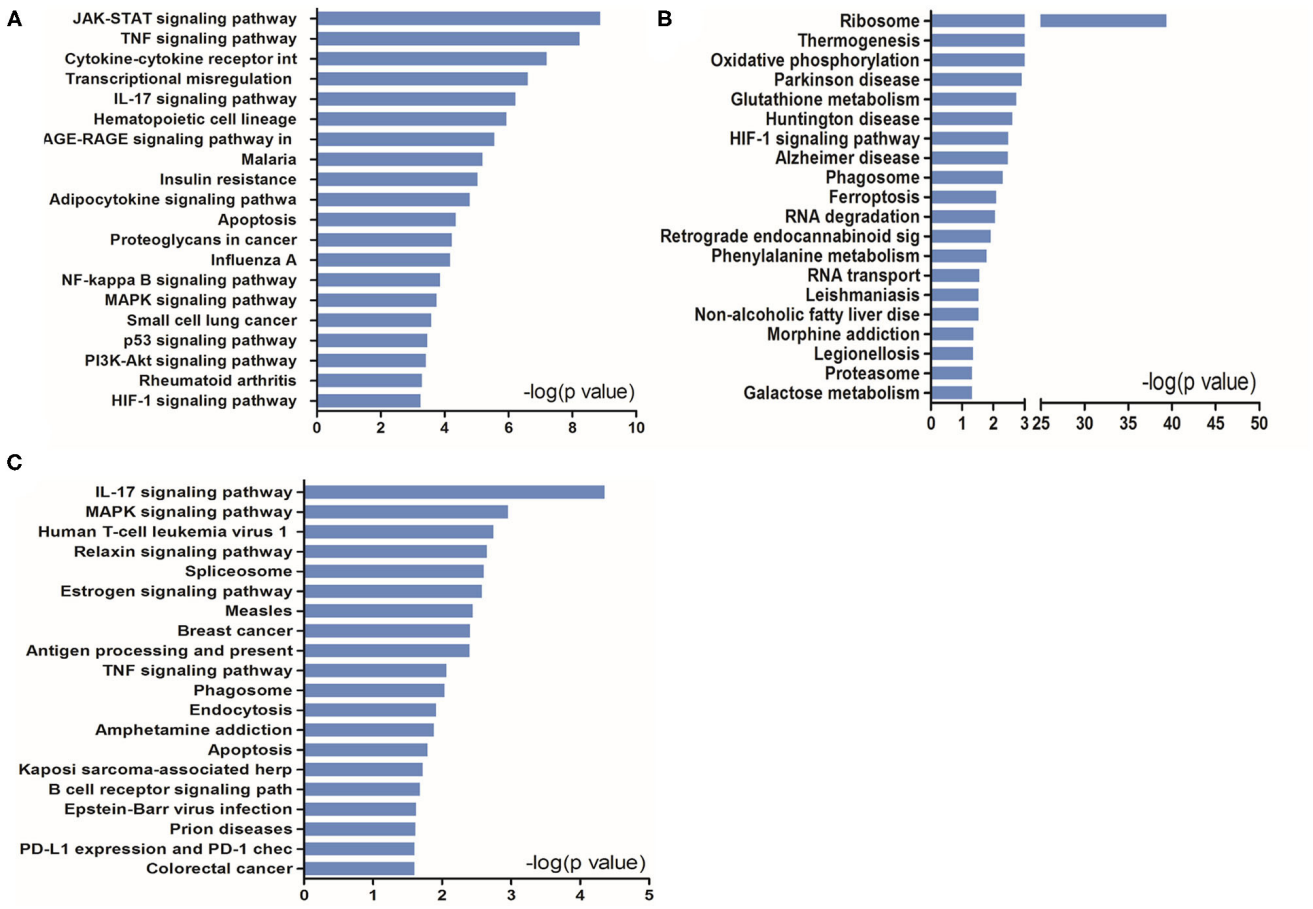
KEGG pathway analysis of up- and downregulated genes in the heart tissues in the presence or absence of *Stat5*. **(A)** The top 20 KEGG pathways were drawn from the 919 differentially expressed genes (DEGs) between the *Stat5*^*fl*/*fl*^+I/R group and the *Stat5*^*fl*/*fl*^+EA+I/R group shown in Figure 5. **(B)** The top 20 KEGG pathways were drawn from the 906 DEGs between the *Stat5-*cKO+I/R group and the *Stat5-*cKO+EA+I/R group shown in Figure 5. **(C)** The top 20 KEGG pathways were drawn from the 133 co-regulated genes shown in Figure 5.

To further understand the potential pathways involved in the regulation of STAT5-related DEGs and that of EAP-related DEGs under conditions of I/R injury, we then carried out a pathway analysis for these DEGs using DAVID Bioinformatics Resources. The top 20 pathways are outlined in Figure 6.

KEGG pathway analysis suggested that, in the presence of *Stat5*, EAP-activated genes were mainly enriched in the JAK/STAT, TNF, IL-17, NF-κB, and MAPK signaling pathways, as well as in cytokine–cytokine receptor interaction (Figure 6A). In contrast, in the *Stat5-*cKO mice, the DEGs associated with EAP-mediated myocardial protection were mainly concentrated in ribosome pathways, thermogenesis, and the oxidative phosphorylation pathway (Figure 6B). We also analyzed the top 20 KEGG pathways associated with the 133 overlapping genes (Figure 6C) and found that some of the EAP-regulated, STAT5-independent DEGs were mainly linked with inflammation-related pathways such as the IL-7 signaling pathway, human T-cell leukemia virus 1 infection, antigen processing and presentation, and the TNF signaling pathway.

### EAP Influenced Apoptotic and Survival Signaling Only in the Presence of STAT5

**Figure 7:**
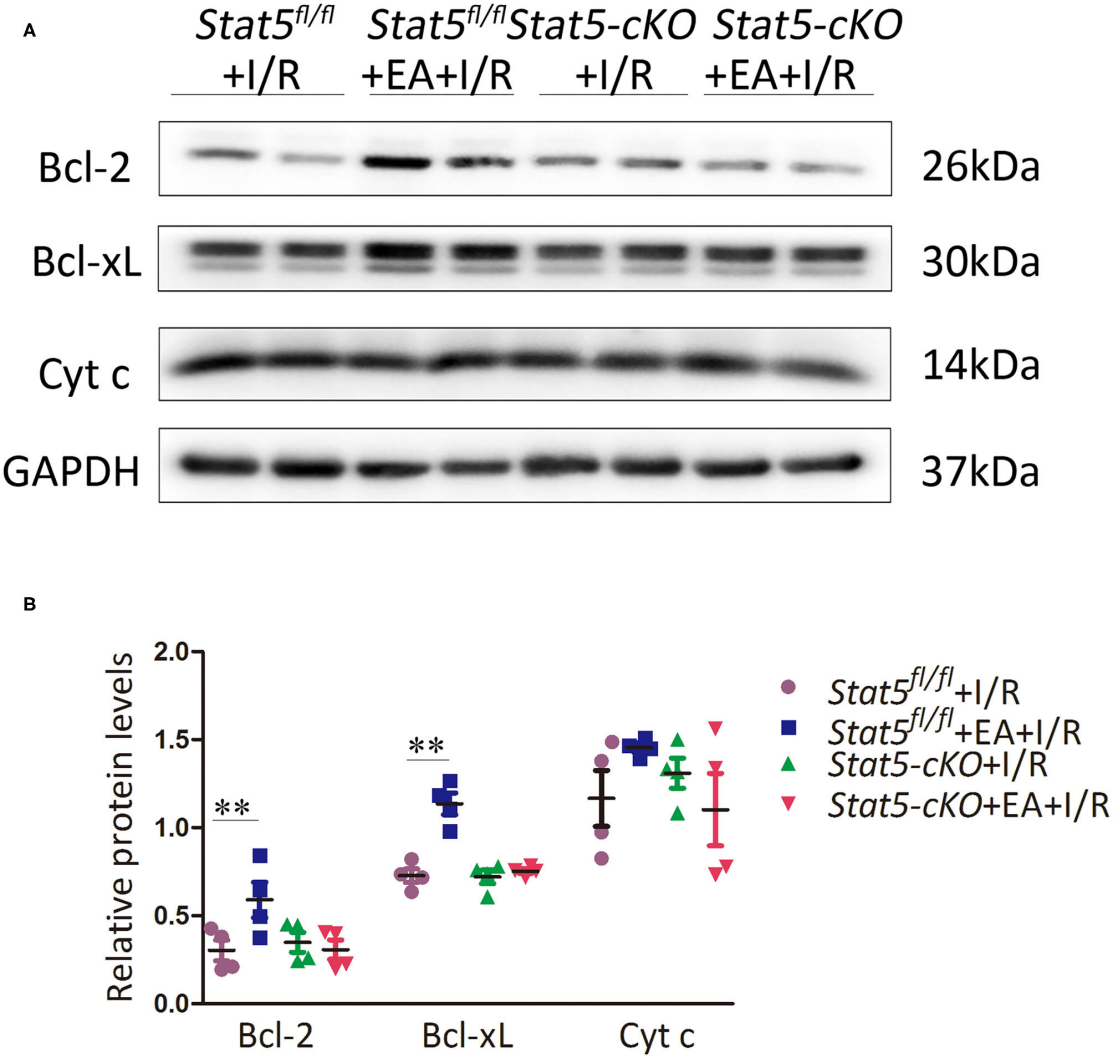
The expression of apoptosis-related proteins. **(A,B)** Western blotting was used to detect the levels of Bcl-2, Bcl-xL, and Cyt c in each group. Data are presented as means ± SEM of more than three independent experiments. **P < 0.01 compared with the *Stat5*^*fl*/*fl*^+I/R group. Data were analyzed by two-way ANOVA with Bonferroni's multiple comparison test, *n* = 4.

**Figure 8:**
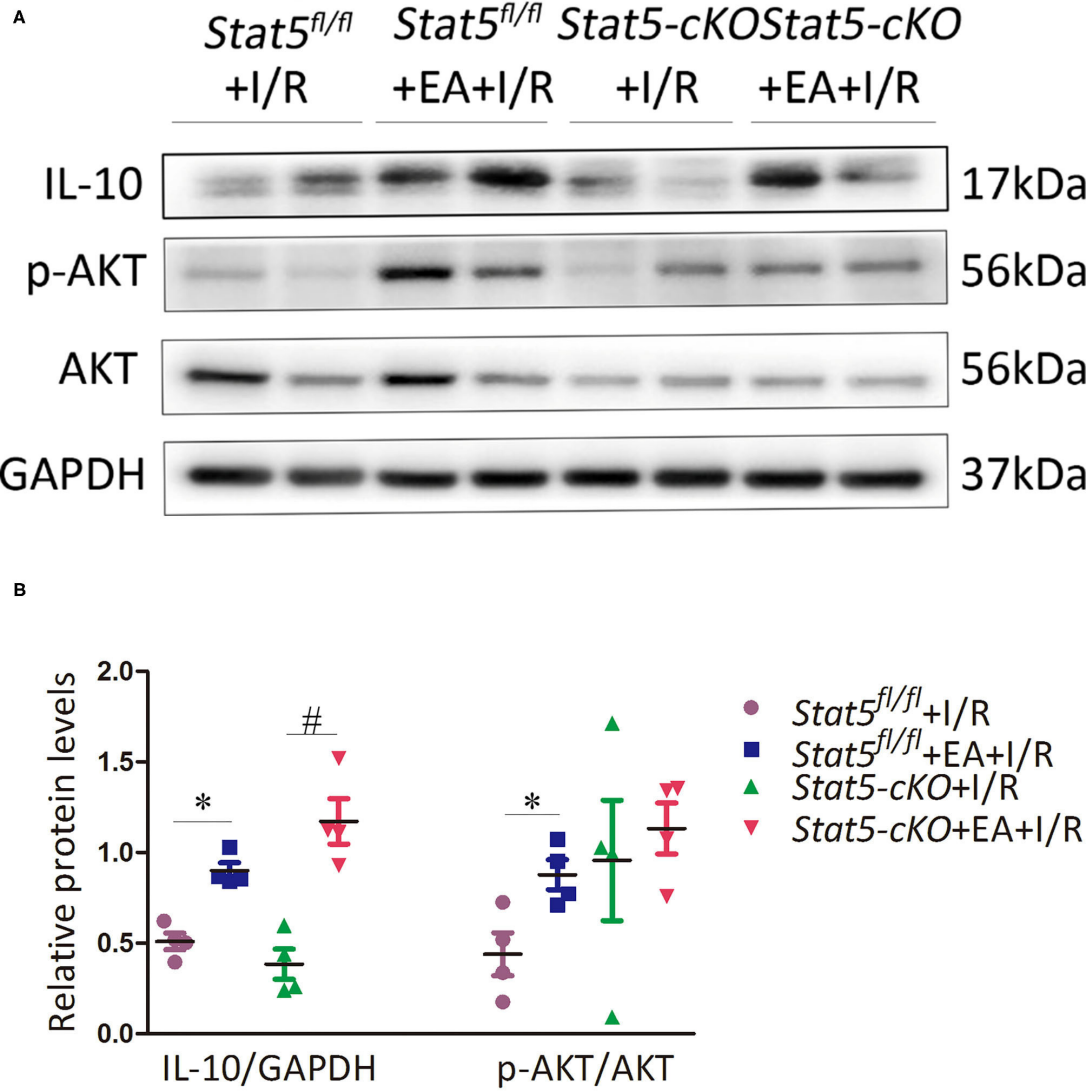
The expression of survival signaling-related proteins. **(A,B)** Western blotting was used to detect the levels of IL-10, p-AKT, and AKT in each group. Data are presented as means ± SEM of more than three independent experiments. *P < 0.05 compared with the *Stat5*^*fl*/*fl*^+I/R group; ^#^*P* < 0.05 compared with the *Stat5-*cKO+I/R group. Data were analyzed by two-way ANOVA with Bonferroni's multiple comparison test, *n* = 4.

The genome-wide profiling data indicated that EAP can activate antiapoptotic and survival signaling in mice with I/R injury. To further validate these findings, we investigated the expression of apoptosis- and survival-related proteins in the myocardial tissue of *Stat5*^*fl*/*fl*^ and *Stat5-*cKO mice following EAP. The results showed that the expression levels of Bcl-2 and Bcl-xL were significantly increased in the *Stat5*^*fl*/*fl*^+EA+I/R group compared with those in the *Stat5*^*fl*/*fl*^+I/R group (*P* < 0.05), whereas the expression of Cyt c did not differ between these groups (Figures 7A,B). In contrast, no marked changes were observed in the expression levels of these proteins in the hearts of *Stat5-*cKO mice either with or without EAP, suggesting that STAT5 is essential for the EAP-mediated activation of antiapoptotic signaling in the I/R injury condition. We then measured the level of IL-10, an important cytokine in cardioprotection, and that of its related proteins PI3K, AKT, and p-AKT (Figures 8A,B). The results showed that EAP increased the levels of p-AKT in the presence, but not absence, of STAT5; however, under the same condition, IL-10 was upregulated in the hearts of both *Stat5*^*fl*/*fl*^ and *Stat5-*cKO mice. These findings suggested that the EAP-induced activation of survival signaling to protect against myocardial I/R injury was partially STAT5-dependent.

## Discussion

Ischemic heart disease remains the leading cause of premature mortality and disability worldwide (34, 42). Although early coronary reperfusion, a clinically effective method against myocardial I/R injury, can reduce infarct size, reperfusion by revascularization initiates a chain reaction that can promote and amplify post-ischemic injury (43, 44). Pretreatment with EA or RIPC represents a valid method of reducing the risk of myocardial injury (3, 6, 45, 46). In our previous study, we found that STAT5 has a significant impact on RIPC-mediated late cardioprotection through regulating antiapoptotic signaling and the PI3K/AKT survival pathway (37). Similar to RIPC, EAP at acupoint PC6 can also help protect the myocardium under certain disease conditions by stimulating multiple functional pathways.

In the present study, we explored the role of STAT5 in EAP-mediated myocardial protection against I/R by employing cardiomyocyte-specific *Stat5-*cKO mice. Surprisingly, we observed that EAP could reduce the infarct size and the levels of myocardial cell apoptosis in both *Stat5*^*fl*/*fl*^ and *Stat5-*cKO mice (Figures 1, 2), suggesting that STAT5 is not indispensable for the cardioprotective effect of EAP against myocardial I/R injury. However, EAP activated STAT5 to promote antiapoptotic and AKT-dependent survival signaling in the presence, but not absence, of *Stat5* (Figures 7A, 8A). This was confirmed by the RNA-seq results for the I/R-injured heart tissues, which showed that STAT5-dependent genes and EAP-regulated genes belonged to different categories (Table 1, Figure 6). Many of the genes regulated by EAP in the presence of *Stat5* (*Stat5*^*fl*/*fl*^+I/R group vs. the *Stat5*^*fl*/*fl*^+EA+I/R group), such as *Fosb, Fos, cxcl1, Cxcl5, Egr1, Egr2, Nr4a3, Socs3, Ccn5, Myl4, Zhx2, Dkk3*, and *Dynll1*, have been reported to play a protective role against myocardial I/R injury, cardiac hypertrophy, or hypoxic insult (47–64). Moreover, in the presence of functional STAT5, many of these genes are known to play antiapoptotic, anti-inflammatory, and antioxidative roles, while some are also involved in STAT3/5 signaling (Table 1A). These DEGs act in many functional pathways, such as the JAK/STAT, TNF, apoptotic, or NF-κB signaling pathways (Figure 6A). Additionally, we found that among the top 30 genes identified as being differentially expressed between the *Stat5-*cKO+EA+I/R and the *Stat5-*cKO+I/R groups when the *Stat5* gene was absent, *Rps6, Mmp3, Pttg1*, and *Rac2* were closely associated with the IL-6/STAT3 signaling pathway, as previously reported (65–74) (Table 1B). Matrix metallopeptidase 3 (*Mmp3*) encodes an extracellular matrix-degrading enzyme (MMP-3) that is closely linked with tissue remodeling, wound repair, and the progression of atherosclerosis (65). Recent findings have indicated that STAT3 binds to the *Mmp3* promoter and promotes its transcription following IL-6 stimulation (75). Pituitary tumor transforming 1 (*Pttg1*) was originally cloned from rat pituitary tumor cells and was reported to function as an oncogene (76). Huang et al. (70) demonstrated that *Pttg1* expression is regulated by IL-6 *via* the binding of activated STAT3 to the *PTTG1* promoter in LNCa P cells. Rac2, a Rac family member, is mainly expressed in hematopoietic cells. Lai et al. detected that Rac can enhance STAT3 activation and regulate the expression of HIF-2α and VEGF, thereby promoting angiogenesis. The same authors also found that the activation of STAT3, but not STAT5, was reduced in Rac-depleted glioblastoma cells. High levels of intracellular galectin-3 expression are essential for the transcriptional activation of osteopontin [OPN; also known as secreted phosphoprotein 1 (Spp1)] in STAT3-mediated macrophage M2 polarization after myocardial infarction (67, 71). The phosphorylation sites on ribosomal protein S6 (Rps6) have been mapped to five clustered residues, which play an important role in protein synthesis in cardiac myocytes, as well as in cardiac function (66, 72–74). Our KEGG pathway analysis indicated that the DEGs activated by EAP in *Stat5-*cKO mice act mainly in ribosome-related, thermogenesis-related, and oxidative phosphorylation-related pathways (Figure 6). Genes involved in the ribosome-related pathway, such as *Rps6* and *Rpl3-ps1*, were markedly upregulated by EAP in mice lacking *Stat5*. Rps6 was reported to be closely related to the IL-6/STAT3 signaling pathway (77, 78). Notably, this pathway has also been linked with mitochondrial function, which is important in cardioprotection (79–81). RNA-seq profiling indicated that the mechanisms underlying the protective effect of EAP against myocardial I/R injury differed between *Stat5*^*fl*/*fl*^ and *Stat5-*cKO mice. Combined with our molecular biological data, these results supported that EAP can activate STAT3 in the absence of *Stat5* and help protect against I/R injury.

Multiple studies have demonstrated that in the absence of a given STAT member, receptors will recruit other STAT members instead (82–87). STAT3 and STAT5 show high homology in their functional domains, and have different effects and underlying mechanisms through binding to distinct loci and regulating specific target genes (88). STAT3 and STAT5 proteins can also bind to the same regulatory oncogenic loci, resulting in compensatory or antagonistic signaling (89, 90). Despite the large number of STAT3/STAT5-related studies, the roles of these two proteins in myocardial I/R injury have not been investigated. Studies have indicated that their roles in cardioprotection may be species-specific (27, 32, 33, 79).

Interestingly, in our study, the level of p-STAT3 was significantly increased in the *Stat5-*cKO+EA+I/R group compared with that in the *Stat5*^*fl*/*fl*^+EA+I/R group (Figure 4), suggesting that EAP activates STAT3, and that this contributed to the protective effect of EAP against myocardium I/R injury in *Stat5-*cKO mice. Furthermore, EAP increased the mRNA expression levels of *gp130* and *Il6* only in *Stat5-*cKO mice (Figure 4B), supporting that IL-6/gp130/STAT3 signaling may be activated to compensate for the loss of *Stat5* following myocardial I/R injury.

Growing evidence has demonstrated the protective role of STAT3 in the heart (30, 32, 79, 91–93). STAT3 helps mitigate cardiac I/R injury by reducing apoptosis or increasing antiapoptotic signaling, upregulating the expression of cardioprotective proteins, decreasing ROS generation, and inhibiting autophagy (92). In addition, the activation of STAT3 is known to enhance mitochondrial function by regulating the transcription of genes encoding proteins such as Bcl-2, Bcl-xL, and VEGF (30, 79, 80, 91). Consistent with these observations, we found that EAP promoted the expression of Bcl-2, Bcl-xL, and p-AKT in *Stat5*^*fl*/*fl*^+I/R mice, which was associated with the activation of IL-6/STAT3 signaling. Notably, IL-10 protein expression was increased in both the *Stat5*^*fl*/*fl*^ and the *Stat5-*cKO mice when EAP was applied followed by I/R injury. IL-10 is an important anti-inflammatory cytokine that can be produced by most cell types, and can affect the growth and differentiation of various hematopoietic cells, as well as increase cell proliferation, angiogenesis, and immune evasion (94, 95). We have previously shown that RIPC can activate the expression of IL-10, p-AKT, Bcl-2, and Bcl-xL, thereby protecting the myocardium (37). Recently, Takahashi et al. (96) showed that IL-22, a member of the IL-10 cytokine family, can activate the myocardial STAT3 signaling pathway and protect against myocardial I/R injury in mice. Other studies have also shown that members of the IL-6 and IL-10 families of cytokines can activate the JAK/STAT3 signaling pathway and induce the transcription of genes involved in cell survival and proliferation (92, 97). In this study, EAP altered the expression of the *Mmp3, Ubb*, and *Myh7* genes, which are closely related to the STAT3 pathway, in *Stat5-*cKO mice with myocardial I/R injury (Table 1B). This suggested that STAT3 may have played a vital cardioprotective role by controlling the expression of these genes, and may also have activated the functions of macrophages and mononuclear phagocytes in its role as a transcriptional regulator of anti-inflammatory-related genes (98–101). Angiogenesis is an indicator of cardioprotection and STAT3 can promote the expression of VEGF, a key angiogenic factor (102, 103). In our study, the expression of VEGFA did not differ among the four groups (Supplementary Figure 1), suggesting that the activation of STAT3 by EAP may not be enough to promote angiogenesis in *Stat5-*cKO mice. Further investigation is needed to clarify this observation.

This study had several limitations. We found that, with EAP, IL-6/gp130/STAT3 signaling was activated in the absence of *Stat5* following I/R injury; however, we did not determine the levels of the associated proteins. Additionally, we did not assess the influence of EAP on mitochondrial function, instead of presenting the apoptotic data alone. The sample size in some experiments was also too small to draw firm conclusions owing to the limited border zone of the heart tissue, even though we pooled 2–3 samples for mRNA extraction to ensure biological duplication. Finally, whole western blots should be presented and not the cut-off pieces.

In summary, in the present study, we demonstrated that EAP can protect against myocardial I/R injury by reducing the myocardial infarct area and activating antiapoptotic, anti-inflammatory, and survival signaling pathways. Although STAT5 is involved in this process, the protective effect of EAP is not STAT5-dependent. STAT3 may compensate for the function of STAT5 in the absence of the *Stat5* gene. Our results suggested that EAP can mimic RIPC but is more effective at protecting the heart against I/R injury.

## Data Availability Statement

The original RNA-seq data in our study are publicly available. This data can be found at the sequence read archive (SRA) in NCBI under the accession number PRJNA738960.

## Ethics Statement

The animal study was reviewed and approved by the Institute for Animal Care and Use Committee at Nanjing University of Chinese Medicine.

## Author Contributions

B-MZ and X-YJ conceived and supervised experiments. H-HG, X-YJ, and B-MZ wrote and edited the manuscript. HC and H-HG performed the experiments and analyzed the data. H-XX carried out the bioinformatic analyses for RNA-seq. All authors contributed to the article and approved the submitted version.

## Conflict of Interest

The authors declare that the research was conducted in the absence of any commercial or financial relationships that could be construed as a potential conflict of interest.

## Supplementary Material

Supplementary Figure 1 The protein expression of VEGFA. Western blotting analysis was used to determine the level of VEGFA in each group. Data are presented as means ± SEM. No differences were found among the four groups. Data were analyzed by two-way ANOVA with Bonferroni's multiple comparison test, *n* = 4.

## References

[1] Tsou MT Huang CH Chiu JH. Electroacupuncture on PC6 (Neiguan) attenuates ischemia/reperfusion injury in rat hearts. Am J Chin Med. (2004) 32:951–65. 10.1142/S0192415X04002557 15673200

[2] Gao J Fu W Jin Z Yu X. A preliminary study on the cardioprotection of acupuncture pretreatment in rats with ischemia and reperfusion: involvement of cardiac beta-adrenoceptors. J Physiol Sci. (2006) 56:275–9. 10.2170/physiolsci.RP006606 16867214

[3] Redington KL Disenhouse T Li J Wei C Dai X Gladstone R. Electroacupuncture reduces myocardial infarct size and improves post-ischemic recovery by invoking release of humoral, dialyzable, cardioprotective factors. J Physiol Sci. (2013) 63:219–23. 10.1007/s12576-013-0259-6 23529221 PMC10717317

[4] Kleinbongard P Skyschally A Heusch G. Cardioprotection by remote ischemic conditioning and its signal transduction. Pflugers Arch. (2017) 469:159–81. 10.1007/s00424-016-1922-6 27928644

[5] Zhao L Li D Zheng H Chang X Cui J Wang R. Acupuncture as adjunctive therapy for chronic stable angina: a randomized clinical trial. JAMA Intern Med. (2019) 179:1388–97. 10.1001/jamainternmed.2019.2407 31355870 PMC6664382

[6] Gao J Fu W Jin Z Yu X. Acupuncture pretreatment protects heart from injury in rats with myocardial ischemia and reperfusion via inhibition of the beta(1)-adrenoceptor signaling pathway. Life Sci. (2007) 80:1484–9. 10.1016/j.lfs.2007.01.019 17303176

[7] Xiao N Li Y Shao ML Cui HF Zhang CY Kong SP. Jiaji (EX-B2)-based electroacupuncture preconditioning attenuates early ischaemia reperfusion injury in the rat myocardium. Evid Based Complement Alternat Med. (2020) 2020:8854033. 10.1155/2020/8854033 33376501 PMC7738790

[8] Liao JM Lin CF Ting H Chang CC Lin YJ Lin TB. Low and high frequency electroacupuncture at Hoku elicits a distinct mechanism to activate sympathetic nervous system in anesthetized rats. Neurosci Lett. (1998) 247:155–8. 10.1016/S0304-3940(98)00298-5 9655616

[9] Chao DM Shen LL Tjen-A-Looi S Pitsillides KF Li P Longhurst JC. Naloxone reverses inhibitory effect of electroacupuncture on sympathetic cardiovascular reflex responses. Am J Physiol. (1999) 276:H2127–34. 10.1152/ajpheart.1999.276.6.H2127 10362696

[10] Longhurst J. Acupuncture's cardiovascular actions: a mechanistic perspective. Med Acupunct. (2013) 25:101–13. 10.1089/acu.2013.0960 24761168 PMC3616410

[11] Zhang J Yong Y Li X Hu Y Wang J Wang YQ. Vagal modulation of high mobility group box-1 protein mediates electroacupuncture-induced cardioprotection in ischemia-reperfusion injury. Sci Rep. (2015) 5:15503. 10.1038/srep15503 26499847 PMC4620449

[12] Huang Y Lu SF Hu CJ Fu SP Shen WX Liu WX. Electro-acupuncture at Neiguan pretreatment alters genome-wide gene expressions and protects rat myocardium against ischemia-reperfusion. Molecules. (2014) 19:16158–78. 10.3390/molecules191016158 25302705 PMC6271995

[13] Zeng Q He H Wang XB Zhou YQ Lin HX Tan ZP. Electroacupuncture preconditioning improves myocardial infarction injury via enhancing AMPK-dependent autophagy in rats. Biomed Res Int. (2018) 2018:1238175. 10.1155/2018/1238175 30175112 PMC6106955

[14] Lu SF Huang Y Wang N Shen WX Fu SP Li Q. Cardioprotective effect of electroacupuncture pretreatment on myocardial ischemia / reperfusion injury via antiapoptotic signaling. Evid Based Complement Alternat Med. (2016) 2016:4609784. 10.1155/2016/4609784 27313648 PMC4897718

[15] Tsai CF Su HH Chen KM Liao JM Yao YT Chen YH. Paeonol protects against myocardial ischemia/reperfusion-induced injury by mediating apoptosis and autophagy crosstalk. Front Pharmacol. (2021) 11:586498. 10.3389/fphar.2020.586498 33551799 PMC7858273

[16] Zhang HX Liu LG Huang GF Zhou L Wu WL Zhang TF. Protective effect of electroacupuncture at the Neiguan point in a rabbit model of myocardial ischemia-reperfusion injury. Can J Cardiol. (2009) 25:359–63. 10.1016/s0828-282x(09)70095-9 19536377 PMC2722479

[17] Ji C Song F Huang G Wang S Liu H Liu S. The protective effects of acupoint gel embedding on rats with myocardial ischemia-reperfusion injury. Life Sci. (2018) 211:51–62. 10.1016/j.lfs.2018.09.010 30195034

[18] Chen J Luo Y Wang S Zhu H Li D. Roles and mechanisms of SUMOylation on key proteins in myocardial ischemia/reperfusion injury. J Mol Cell Cardiol. (2019) 134:154–64. 10.1016/j.yjmcc.2019.07.009 31344368

[19] Dai QF Gao JH Xin JJ Liu Q Jing XH Yu XC. The role of adenosine A2b receptor in mediating the cardioprotection of electroacupuncture pretreatment via influencing Ca key regulators. Evid Based Complement Alternat Med. (2019) 2019:6721286. 10.1155/2019/6721286 31885657 PMC6925712

[20] Zhang T Yang WX Wang YL Yuan J Qian Y Sun QM. Electroacupuncture preconditioning attenuates acute myocardial ischemia injury through inhibiting NLRP3 inflammasome activation in mice. Life Sci. (2020) 248:117451. 10.1016/j.lfs.2020.117451 32088213

[21] Fu SP He SY Xu B Hu CJ Lu SF Shen WX. Acupuncture promotes angiogenesis after myocardial ischemia through H3K9 acetylation regulation at VEGF gene. PLoS ONE. (2014) 9:e94604. 10.1371/journal.pone.0094604 24722278 PMC3983235

[22] Zhang J Zhu L Li H Tang Q. Electroacupuncture pretreatment as a novel avenue to protect heart against ischemia and reperfusion injury. Evid Based Complement Alternat Med. (2020) 2020:9786482. 10.1155/2020/9786482 32508960 PMC7254080

[23] Xiao Y Chen W Zhong Z Ding L Bai H Chen H. Electroacupuncture preconditioning attenuates myocardial ischemia-reperfusion injury by inhibiting mitophagy mediated by the mTORC1-ULK1-FUNDC1 pathway. Biomed Pharmacother. (2020) 127:110148. 10.1016/j.biopha.2020.110148 32344255

[24] Heusch G Bøtker HE Przyklenk K Redington A Yellon D. Remote ischemic conditioning. J Am Coll Cardiol. (2015) 65:177–95. 10.1016/j.jacc.2014.10.031 25593060 PMC4297315

[25] Heusch G. Molecular basis of cardioprotection: signal transduction in ischemic pre-, post-, remote conditioning. Circ Res. (2015) 116:674–99. 10.1161/CIRCRESAHA.116.305348 25677517

[26] Merlocco AC Redington KL Disenhouse T Strantza SC Gladstone R Wei C. Transcutaneous electrical nerve stimulation as a novel method of remote preconditioning: *in vitro* validation in an animal model and first human observations. Basic Res Cardiol. (2014) 109:406. 10.1007/s00395-014-0406-0 24604614

[27] Heusch G Musiolik J Kottenberg E Peters J Jakob H Thielmann M. STAT5 activation and cardioprotection by remote ischemic preconditioning in humans: short communication. Circ Res. (2012) 110:111–5. 10.1161/CIRCRESAHA.111.259556 22116817

[28] Skyschally A Gent S Amanakis G Schulte C Kleinbongard P Heusch G. Across-species transfer of protection by remote ischemic preconditioning with species-specific myocardial signal transduction by reperfusion injury salvage kinase and survival activating factor enhancement pathways. Circ Res. (2015) 117:279–88. 10.1161/CIRCRESAHA.117.306878 26058828

[29] Hausenloy DJ Barrabes JA Bøtker HE Davidson SM Di Lisa F Downey J. Ischaemic conditioning and targeting reperfusion injury: a 30 year voyage of discovery. Basic Res Cardiol. (2016) 111:70. 10.1007/s00395-016-0588-8 27766474 PMC5073120

[30] Hildebrandt HA Kreienkamp V Gent S Kahlert P Heusch G Kleinbongard P. Kinetics and signal activation properties of circulating factor(s) from healthy volunteers undergoing remote ischemic pre-conditioning. JACC Basic Transl Sci. (2016) 1:3–13. 10.1016/j.jacbts.2016.01.007 27642642 PMC5012372

[31] Lieder HR Kleinbongard P Skyschally A Hagelschuer H Chilian WM Heusch G. Vago-splenic axis in signal transduction of remote ischemic preconditioning in pigs and rats. Circ Res. (2018) 123:1152–63. 10.1161/CIRCRESAHA.118.313859 30359199 PMC7304918

[32] Skyschally A Kleinbongard P Lieder H Gedik N Stoian L Amanakis G. Humoral transfer and intramyocardial signal transduction of protection by remote ischemic preconditioning in pigs, rats, and mice. Am J Physiol Heart Circ Physiol. (2018) 315:H159–72. 10.1152/ajpheart.00152.2018 29569956

[33] Wu Q Wang T Chen S Zhou Q Li H Hu N. Cardiac protective effects of remote ischaemic preconditioning in children undergoing tetralogy of fallot repair surgery: a randomized controlled trial. Eur Heart J. (2018) 39:1028–37. 10.1093/eurheartj/ehx030 28329231 PMC6018784

[34] Heusch G. Myocardial ischaemia-reperfusion injury and cardioprotection in perspective. Nat Rev Cardiol. (2020) 17:773–89. 10.1038/s41569-020-0403-y 32620851

[35] Milani-Nejad N Janssen PM. Small and large animal models in cardiac contraction research: advantages and disadvantages. Pharmacol Ther. (2014) 141:235–49. 10.1016/j.pharmthera.2013.10.007 24140081 PMC3947198

[36] Heusch G Gersh BJ. The pathophysiology of acute myocardial infarction and strategies of protection beyond reperfusion: a continual challenge. Eur Heart J. (2017) 38:774–84. 10.1093/eurheartj/ehw224 27354052

[37] Chen H Jing XY Shen YJ Wang TL Ou C Lu SF. Stat5-dependent cardioprotection in late remote ischemia preconditioning. Cardiovasc Res. (2018) 114:679–89. 10.1093/cvr/cvy014 29365089

[38] Ye Y Birnbaum Y Widen SG Zhang Z Zhu S Bajaj M. Acupuncture reduces hypertrophy and cardiac fibrosis, and improves heart function in mice with diabetic cardiomyopathy. Cardiovasc Drugs Ther. (2020) 34:835–48. 10.1007/s10557-020-07043-4 32767170

[39] Gao E Boucher M Chuprun JK Zhou RH Eckhart AD Koch WJ. Darbepoetin alfa, a long-acting erythropoietin analog, offers novel and delayed cardioprotection for the ischemic heart. Am J Physiol Heart Circ Physiol. (2007) 293:H60–8. 10.1152/ajpheart.00227.2007 17384131

[40] Gao E Lei YH Shang X Huang ZM Zuo L Boucher M. A novel and efficient model of coronary artery ligation and myocardial infarction in the mouse. Circ Res. (2010) 107:1445–53. 10.1161/CIRCRESAHA.110.223925 20966393 PMC3005817

[41] Fu SP Hong H Lu SF Hu CJ Xu HX Li Q. Genome-wide regulation of electro-acupuncture on the neural Stat5-loss-induced obese mice. PLoS ONE. (2017) 12:e0181948. 10.1371/journal.pone.0181948 28806763 PMC5555711

[42] Gupta R Wood DA. Primary prevention of ischaemic heart disease: populations, individuals, health professionals. Lancet. (2019) 394:685–96. 10.1016/S0140-6736(19)31893-8 31448740

[43] Yellon DM Hausenloy DJ. Myocardial reperfusion injury. N Engl J Med. (2007) 357:1121–35. 10.1056/NEJMra071667 17855673

[44] Binder A Ali A Chawla R Aziz HA Abbate A Jovin IS. Myocardial protection from ischemia-reperfusion injury post coronary revascularization. Expert Rev Cardiovasc Ther. (2015) 13:1045–57. 10.1586/14779072.2015.1070669 26202544

[45] Hausenloy DJ Kharbanda RK Møller UK Ramlall M Aarøe J Butler R. Effect of remote ischaemic conditioning on clinical outcomes in patients with acute myocardial infarction (CONDI-2/ERIC-PPCI): a single-blind randomised controlled trial. Lancet. (2019) 394:1415–24. 10.1016/S0140-6736(19)32039-2 31500849 PMC6891239

[46] Kepler T Kuusik K Lepner U Starkopf J Zilmer M Eha J. Remote ischaemic preconditioning attenuates cardiac biomarkers during vascular surgery: a randomised clinical trial. Eur J Vasc Endovasc Surg. (2020) 59:301–8. 10.1016/j.ejvs.2019.09.502 31870692

[47] Alam MJ Gupta R Mahapatra NR Goswami SK. Catestatin reverses the hypertrophic effects of norepinephrine in H9c2 cardiac myoblasts by modulating the adrenergic signaling. Mol Cell Biochem. (2020) 464:205–19. 10.1007/s11010-019-03661-1 31792650

[48] Alfonso-Jaume MA Bergman MR Mahimkar R Cheng S Jin ZQ Karliner JS. Cardiac ischemia-reperfusion injury induces matrix metalloproteinase-2 expression through the AP-1 components FosB and JunB. Am J Physiol Heart Circ Physiol. (2006) 291:H1838–46. 10.1152/ajpheart.00026.2006 16699069

[49] Udoko AN Johnson CA Dykan A Rachakonda G Villalta F Mandape SN. Early regulation of profibrotic genes in primary human cardiac myocytes by Trypanosoma cruzi. PLoS Negl Trop Dis. (2016) 10:e0003747. 10.1371/journal.pntd.0003747 26771187 PMC4714843

[50] Sulston R Kelly V Walker BR Porter KE Chapman KE Gray GA. 11β-HSD1 suppresses cardiac fibroblast CXCL2, CXCL5 and neutrophil recruitment to the heart post MI. J. Endocrinol. (2017) 233, 315–327. 10.1530/JOE-16-0501 28522730 PMC5457506

[51] Tang Y Wang Y Park KM Hu Q Teoh JP Broskova Z. MicroRNA-150 protects the mouse heart from ischaemic injury by regulating cell death. Cardiovasc Res. (2015) 106:387–97. 10.1093/cvr/cvv121 25824147 PMC4447807

[52] Jiang Y Feng YP Tang LX Yan YL Bai JW. The protective role of NR4A3 in acute myocardial infarction by suppressing inflammatory responses via JAK2-STAT3/NF-βB pathway. Biochem Biophys Res Commun. (2019) 517:697–702. 10.1016/j.bbrc.2019.07.116 31399192

[53] Saddic LA Howard-Quijano K Kipke J Kubo Y Dale EA Hoover D. Progression of myocardial ischemia leads to unique changes in immediate-early gene expression in the spinal cord dorsal horn. Am J Physiol Heart Circ Physiol. (2018) 315:H1592–601. 10.1152/ajpheart.00337.2018 30216122 PMC6336975

[54] Bos JM Subramaniam M Hawse JR Christiaans I Rajamannan NM Maleszewski JJ. TGFβ-inducible early gene-1 (TIEG1) mutations in hypertrophic cardiomyopathy. J Cell Biochem. (2012) 113:1896–903. 10.1002/jcb.24058 22234868 PMC3495561

[55] Oba T Yasukawa H Hoshijima M Sasaki K Futamata N Fukui D. Cardiac-specific deletion of SOCS-3 prevents development of left ventricular remodeling after acute myocardial infarction. J Am Coll Cardiol. (2012) 59:838–52. 10.1016/j.jacc.2011.10.887 22361405

[56] Stobdan T Zhou D Ao-Ieong E Ortiz D Ronen R Hartley I. Endothelin receptor B, a candidate gene from human studies at high altitude, improves cardiac tolerance to hypoxia in genetically engineered heterozygote mice. Proc Natl Acad Sci USA. (2015) 112:10425–30. 10.1073/pnas.1507486112 26240367 PMC4547246

[57] El-Magd MA Abdo WS El-Maddaway M Nasr NM Gaber RA El-Shetry ES. High doses of S-methylcysteine cause hypoxia-induced cardiomyocyte apoptosis accompanied by engulfment of mitochondria by nucleus. Biomed Pharmacother. (2017) 94:589–97. 10.1016/j.biopha.2017.07.100 28783581

[58] Wu JB Zhou Y Liang CL Zhang XJ Lai JM Ye SF. Cyclovirobuxinum D alleviates cardiac hypertrophy in hyperthyroid rats by preventing apoptosis of cardiac cells and inhibiting the p38 mitogen-activated protein kinase signaling pathway. Chin J Integr Med. (2017) 23:770–8. 10.1007/s11655-015-2299-7 27048408

[59] Wang XT Wu XD Lu YX Sun YH Zhu HH Liang JB. Egr-1 is involved in coronary microembolization-induced myocardial injury via Bim/Beclin-1 pathway-mediated autophagy inhibition and apoptosis activation. Aging. (2018) 10:3136–47. 10.18632/aging.101616 30391937 PMC6286823

[60] Zhai CG Xu YY Tie YY Zhang Y Chen WQ Ji XP. DKK3 overexpression attenuates cardiac hypertrophy and fibrosis in an angiotensin-perfused animal model by regulating the ADAM17/ACE2 and GSK-3β/β-catenin pathways. J Mol Cell Cardiol. (2018) 114:243–52. 10.1016/j.yjmcc.2017.11.018 29196099

[61] Kubota A Suto A Suzuki K Kobayashi Y Nakajima H. Matrix metalloproteinase-12 produced by Ly6C macrophages prolongs the survival after myocardial infarction by preventing neutrophil influx. J Mol Cell Cardiol. (2019) 131:41–52. 10.1016/j.yjmcc.2019.04.007 31009606

[62] Ma P Li Y Wang S Wang G Yan C Li Z. SOCS3 promotes myocardial cell apoptosis in myocardial ischemia reperfusion rats via JAK/STAT signaling pathway. Minerva Cardioangiol. (2020) 68:164–6. 10.23736/S0026-4725.19.05046-1 32030968

[63] Sun S Cui Z Yan T Wu J Liu ZH. CCN5 inhibits proliferation and promotes apoptosis of oral squamous cell carcinoma cells. Cell Biol Int. (2020) 44:998–1008. 10.1002/cbin.11296 31889370

[64] Tian X Wang Y Li S Yue W Tian H. ZHX2 inhibits proliferation and promotes apoptosis of human lung cancer cells through targeting p38MAPK pathway. Cancer Biomark. (2020) 27:75–84. 10.3233/CBM-190514 31683461 PMC12662273

[65] Abilleira S Bevan S Markus HS. The role of genetic variants of matrix metalloproteinases in coronary and carotid atherosclerosis. J Med Genet. (2006) 43:897–901. 10.1136/jmg.2006.040808 16905683 PMC2563195

[66] Calamaras TD Lee C Lan F Ido Y Siwik DA Colucci WS. The lipid peroxidation product 4-hydroxy-trans-2-nonenal causes protein synthesis in cardiac myocytes via activated mTORC1-p70S6K-RPS6 signaling. Free Radic Biol Med. (2015) 82:137–46. 10.1016/j.freeradbiomed.2015.01.007 25617592 PMC4387097

[67] Wen Y Feng D Wu H Liu W Li H Wang F. Defective initiation of liver regeneration in osteopontin-deficient mice after partial hepatectomy due to insufficient activation of IL-6/Stat3 pathway. Int J Biol Sci. (2015) 11:1236–47. 10.7150/ijbs.12118 26327817 PMC4551759

[68] Lai YJ Tsai JC Tseng YT Wu MS Liu WS Lam HI. Small G protein Rac GTPases regulate the maintenance of glioblastoma stem-like cells *in vitro* and *in vivo*. Oncotarget. (2017) 8:18031–49. 10.18632/oncotarget.14949 28160553 PMC5392305

[69] Zhu XM Sun WF. Association between matrix metalloproteinases polymorphisms and ovarian cancer risk: a meta-analysis and systematic review. PLoS ONE. (2017) 12:e0185456. 10.1371/journal.pone.0185456 28957437 PMC5619784

[70] Huang S Liu Q Liao Q Wu Q Sun B Yang Z. Interleukin-6/signal transducer and activator of transcription 3 promotes prostate cancer resistance to androgen deprivation therapy via regulating pituitary tumor transforming gene 1 expression. Cancer Sci. (2018) 109:678–87. 10.1111/cas.13493 29288516 PMC5834804

[71] Shirakawa K Endo J Kataoka M Katsumata Y Yoshida N Yamamoto T. IL (Interleukin)-10-STAT3-galectin-3 axis is essential for osteopontin-producing reparative macrophage polarization after myocardial infarction. Circulation. (2018) 138:2021–35. 10.1161/CIRCULATIONAHA.118.035047 29967195

[72] Sharma S Mazumder AG Rana AK Patial V Singh D. Spontaneous recurrent seizures mediated cardiac dysfunction via mTOR pathway upregulation: a putative target for SUDEP management. CNS Neurol Disord Drug Targets. (2019) 18:555–65. 10.2174/1871527318666190801112027 31368880

[73] Dern K Burns TA Watts MR van Eps AW Belknap JK. Influence of digital hypothermia on lamellar events related to IL-6/gp130 signalling in equine sepsis-related laminitis. Equine Vet J. (2020) 52:441–8. 10.1111/evj.13184 31509270

[74] Wu RM Jiang B Li H Dang WZ Bao WL Li HD. A network pharmacology approach to discover action mechanisms of Yangxinshi Tablet for improving energy metabolism in chronic ischemic heart failure. J Ethnopharmacol. (2020) 246:112227. 10.1016/j.jep.2019.112227 31509780

[75] Araki Y Tsuzuki WT Aizaki Y Sato K Yokota K Fujimoto K. Histone methylation and STAT-3 differentially regulate interleukin-6-induced matrix metalloproteinase gene activation in rheumatoid arthritis synovial fibroblasts. Arthritis Rheumatol. (2016) 68:1111–23. 10.1002/art.39563 26713842

[76] Asari Y Kageyama K Nakada Y Tasso M Takayasu S Niioka K. Inhibitory effects of a selective Jak2 inhibitor on adrenocorticotropic hormone production and proliferation of corticotroph tumor AtT20 cells. Onco Targets Ther. (2017) 10:4329–38. 10.2147/OTT.S141345 28919782 PMC5590765

[77] Meyuhas O. Ribosomal protein S6 phosphorylation: four decades of research. Int Rev Cell Mol Biol. (2015) 320:41–73. 10.1016/bs.ircmb.2015.07.006 26614871

[78] Gopinath SD. Inhibition of Stat3 signaling ameliorates atrophy of the soleus muscles in mice lacking the vitamin D receptor. Skelet Muscle. (2017) 7:2. 10.1186/s13395-017-0121-2 28122601 PMC5264327

[79] Boengler K Buechert A Heinen Y Roeskes C Hilfiker-Kleiner D Heusch G. Cardioprotection by ischemic postconditioning is lost in aged and STAT3-deficient mice. Circ Res. (2008) 102:131–5. 10.1161/CIRCRESAHA.107.164699 17967780

[80] Lecour S. Activation of the protective Survivor Activating Factor Enhancement (SAFE) pathway against reperfusion injury: does it go beyond the RISK pathway? J Mol Cell Cardiol. (2009) 47:32–40. 10.1016/j.yjmcc.2009.03.019 19344728

[81] Beak JY Kang HS Huang W Myers PH Bowles DE Jetten AM. The nuclear receptor RORα protects against angiotensin II-induced cardiac hypertrophy and heart failure. Am J Physiol Heart Circ Physiol. (2019) 316:H186–200. 10.1152/ajpheart.00531.2018 30387679 PMC6383360

[82] Hennighausen L Robinson GW. Interpretation of cytokine signaling through the transcription factors STAT5A and STAT5B. Genes Dev. (2008) 22:711–21. 10.1101/gad.1643908 18347089 PMC2394721

[83] Hosui A Kimura A Yamaji D Zhu BM Na R Hennighausen L. Loss of STAT5 causes liver fibrosis and cancer development through increased TGF-{beta} and STAT3 activation. J Exp Med. (2009) 206:819–31. 10.1084/jem.20080003 19332876 PMC2715112

[84] Yu JH Zhu BM Wickre M Riedlinger G Chen W Hosui A. The transcription factors signal transducer and activator of transcription 5A (STAT5A) and STAT5B negatively regulate cell proliferation through the activation of cyclin-dependent kinase inhibitor 2b (Cdkn2b) and Cdkn1a expression. Hepatology. (2010) 52:1808–18. 10.1002/hep.23882 21038417 PMC3152209

[85] Friedbichler K Themanns M Mueller KM Schlederer M Kornfeld JW Terracciano LM. Growth-hormone-induced signal transducer and activator of transcription 5 signaling causes gigantism, inflammation, and premature death but protects mice from aggressive liver cancer. Hepatology. (2012) 55:941–52. 10.1002/hep.24765 22031092

[86] Valle-Mendiola A Soto-Cruz I. Energy metabolism in cancer: the roles of STAT3 and STAT5 in the regulation of metabolism-related genes. Cancers. (2020) 12:124. 10.3390/cancers12010124 31947710 PMC7016889

[87] Hin Tang JJ Thng DKH Lim JJ Toh TB. JAK/STAT signaling in hepatocellular carcinoma. Hepat Oncol. (2020) 7:HEP18. 10.2217/hep-2020-0001 32273976 PMC7137178

[88] Wingelhofer B Neubauer HA Valent P Han X Constantinescu SN Gunning PT. Implications of STAT3 and STAT5 signaling on gene regulation and chromatin remodeling in hematopoietic cancer. Leukemia. (2018) 32:1713–26. 10.1038/s41375-018-0117-x 29728695 PMC6087715

[89] Walker SR Nelson EA Yeh JE Pinello L Yuan GC Frank DA. STAT5 outcompetes STAT3 to regulate the expression of the oncogenic transcriptional modulator BCL6. Mol Cell Biol. (2013) 33:2879–90. 10.1128/MCB.01620-12 23716595 PMC3719667

[90] Walker SR Xiang M Frank DA. Distinct roles of STAT3 and STAT5 in the pathogenesis and targeted therapy of breast cancer. Mol Cell Endocrinol. (2014) 382:616–21. 10.1016/j.mce.2013.03.010 23531638 PMC3732813

[91] Heusch G Musiolik J Gedik N Skyschally A. Mitochondrial STAT3 activation and cardioprotection by ischemic postconditioning in pigs with regional myocardial ischemia/reperfusion. Circ Res. (2011) 109:1302–8. 10.1161/CIRCRESAHA.111.255604 21980124

[92] Harhous Z Booz GW Ovize M Bidaux G Kurdi M. An update on the multifaceted roles of STAT3 in the heart. Front Cardiovasc Med. (2019) 6:150. 10.3389/fcvm.2019.00150 31709266 PMC6823716

[93] Nakao S Tsukamoto T Ueyama T Kawamura T. STAT3 for cardiac regenerative medicine: involvement in stem cell biology, pathophysiology, and bioengineering. Int J Mol Sci. (2020) 21:1937. 10.3390/ijms21061937 32178385 PMC7139789

[94] Hodge DR Hurt EM Farrar WL. The role of IL-6 and STAT3 in inflammation and cancer. Eur J Cancer. (2005) 41:2502–12. 10.1016/j.ejca.2005.08.016 16199153

[95] Zhang Z Yao L Yang J Wang Z Du G. PI3K/Akt and HIF-1 signaling pathway in hypoxia-ischemia (review). Mol Med Rep. (2018) 18:3547–54. 10.3892/mmr.2018.9375 30106145 PMC6131612

[96] Takahashi J Yamamoto M Yasukawa H Nohara S Nagata T Shimozono K. Interleukin-22 directly activates myocardial STAT3 (Signal Transducer and Activator of Transcription-3) signaling pathway and prevents myocardial ischemia reperfusion injury. J Am Heart Assoc. (2020) 9:e014814. 10.1161/JAHA.119.014814 32301368 PMC7428538

[97] Huynh J Etemadi N Hollande F Ernst M Buchert M. The JAK/STAT3 axis: a comprehensive drug target for solid malignancies. Semin Cancer Biol. (2017) 45:13–22. 10.1016/j.semcancer.2017.06.001 28647610

[98] Donnelly RP Dickensheets H Finbloom DS. The interleukin-10 signal transduction pathway and regulation of gene expression in mononuclear phagocytes. J Interferon Cytokine Res. (1999) 19:563–73. 10.1089/107999099313695 10433356

[99] Lang R. Tuning of macrophage responses by Stat3-inducing cytokines: molecular mechanisms and consequences in infection. Immunobiology. (2005) 210:63–76. 10.1016/j.imbio.2005.05.001 16164013

[100] Murray PJ. Understanding and exploiting the endogenous interleukin-10/STAT3-mediated anti-inflammatory response. Curr Opin Pharmacol. (2006) 6:379–86. 10.1016/j.coph.2006.01.010 16713356

[101] Schmetterer KG Pickl WF. The IL-10/STAT3 axis: Contributions to immune tolerance by thymus and peripherally derived regulatory T-cells. Eur J Immunol. (2017) 47:1256–65. 10.1002/eji.201646710 28631311

[102] Funamoto M Fujio Y Kunisada K Negoro S Tone E Osugi T. Signal transducer and activator of transcription 3 is required for glycoprotein 130-mediated induction of vascular endothelial growth factor in cardiac myocytes. J Biol Chem. (2000) 275:10561–6. 10.1074/jbc.275.14.10561 10744750

[103] Osugi T Oshima Y Fujio Y Funamoto M Yamashita A Negoro S. Cardiac-specific activation of signal transducer and activator of transcription 3 promotes vascular formation in the heart. J Biol Chem. (2002) 277:6676–81. 10.1074/jbc.M108246200 11744720

