# Supplementary figures and images for: STAT3 but Not STAT5 Contributes to the Protective Effect of Electroacupuncture Against Myocardial Ischemia/Reperfusion Injury in Mice
Source: Front Med (Lausanne). 2021 Jul 9;8:649654. doi: 10.3389/fmed.2021.649654 (PMC8299366; doi:10.3389/fmed.2021.649654)

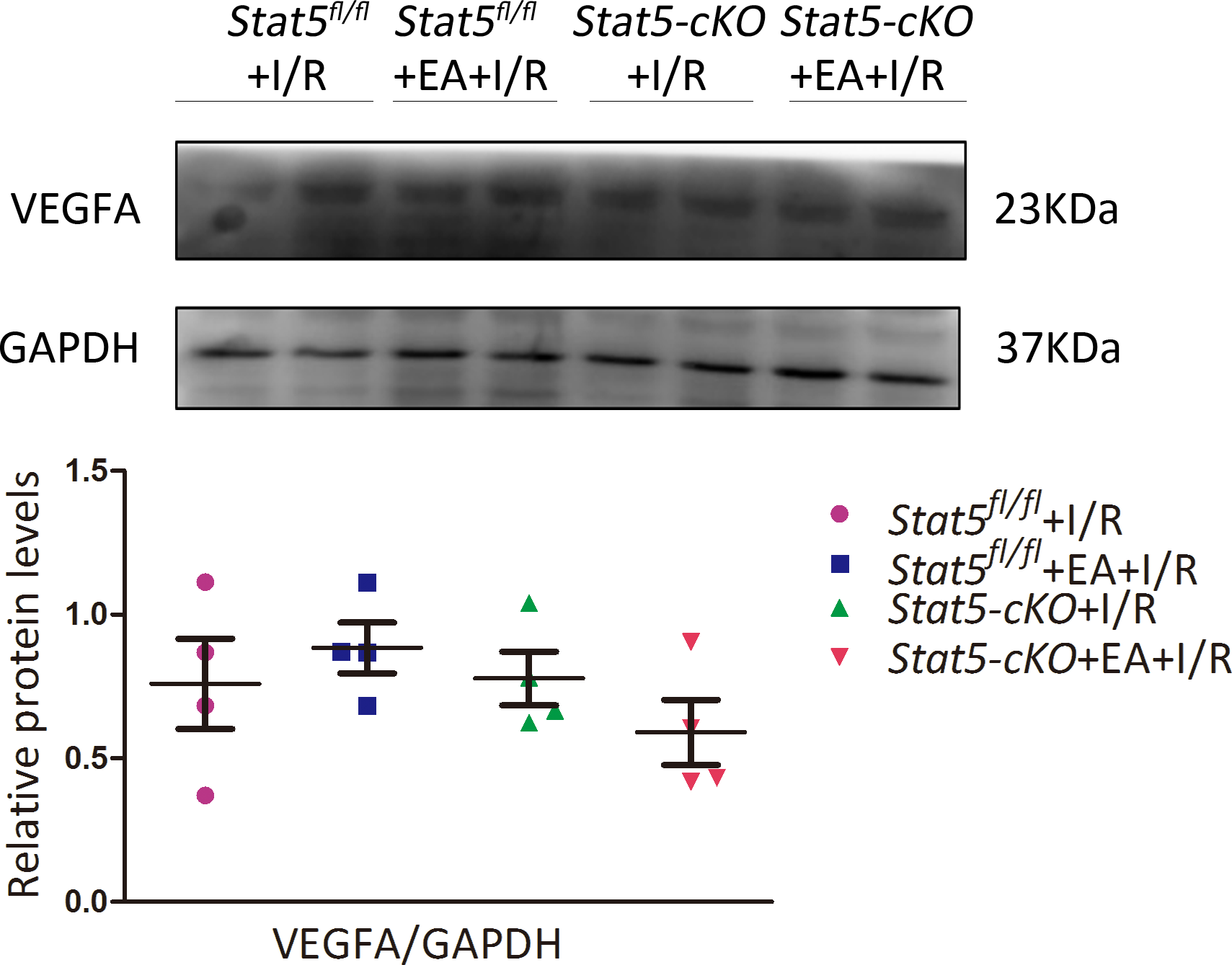

Supplement: Supplementary Figure 1 — The protein expression of VEGFA. Western blotting analysis was used to determine the level of VEGFA in each group. Data are presented as means ± SEM. No differences were found among the four groups. Data were analyzed by two-way ANOVA with Bonferroni's multiple comparison test, n = 4. [file Image_1.TIF]
